# Dysregulated Redox Signaling and Its Impact on Inflammatory Pathways, Mitochondrial Dysfunction, Autophagy and Cardiovascular Diseases

**DOI:** 10.3390/antiox14111278

**Published:** 2025-10-24

**Authors:** Mehnaz Pervin, Judy B. de Haan

**Affiliations:** 1 Cardiovascular Inflammation and Redox Biology Laboratory, Baker Heart and Diabetes Institute, 75 Commercial Road, Melbourne, VIC 3004, Australia; mehnaz.pervin@baker.edu.au; 2 Department of Diabetes, School of Translational Medicine, Monash University, Melbourne, VIC 3004, Australia; 3 Department of Immunology and Pathology, School of Translational Medicine, Monash University, Melbourne, VIC 3004, Australia; 4 Department of Physiology, Anatomy and Microbiology, La Trobe University, Bundoora, VIC 3083, Australia; 5 Department of Chemistry and Biotechnology, Swinburne University of Technology, Hawthorn, VIC 3122, Australia; 6 Baker Department of Cardiometabolic Health, Melbourne University, Melbourne, VIC 3010, Australia

**Keywords:** redox signaling, oxidative stress, mitochondrial dysfunction, autophagy, inflammation, cardiovascular disease

## Abstract

Dysregulated redox signaling, mitochondrial dysfunction and impaired autophagy form an interconnected network that drives inflammatory and immune responses in cardiovascular disease. Among these, disturbances in redox balance, largely mediated by reactive oxygen species (ROS), serve as key drivers linking inflammatory signaling to adverse cardiovascular outcomes. Mitochondria are essential for energy production and cellular homeostasis, but their dysfunction leads to the accumulation of excessive ROS, which triggers inflammation. This pro-oxidative milieu disrupts immune regulation by activating inflammasomes, promoting cytokine secretion, triggering immune cell infiltration and ultimately contributing to cardiovascular injury. Conversely, intracellular degradation processes such as mitophagy alleviate these effects by selectively eliminating dysfunctional mitochondria, thereby decreasing ROS levels and maintaining immune homoeostasis. These interconnected processes influence myeloid cell function, including mitochondrial reprogramming, macrophage polarization and autophagic activity. The modulation of these immune responses is crucial for determining the severity and resolution of cardiac and vascular inflammation, and consequently the extent of cellular injury. This review examines the latest developments and understanding of the intricate relationships between redox signaling, mitochondrial dysfunction, autophagy and oxidative stress in modulating inflammation and immune responses in cardiovascular diseases. Understanding these interrelationships will inform future studies and therapeutic solutions for the prevention and treatment of cardiovascular diseases.

## 1. Introduction

Cardiovascular disease (CVD) remains a major public health crisis worldwide, and its prevalence is expected to rise over the next few decades [1,2]. A substantial risk of CVD-related mortality persists despite current conventional therapies, such as statins, angiotensin II-converting enzyme (ACE) inhibitors, angiotensin receptor blockers (ARBs), calcium channel blockers, beta-blockers and lifestyle modifications, particularly in patients with obesity, diabetes and chronic inflammation. The global number of cardiovascular deaths is estimated to increase from 20.5 million in 2025 to approximately 35.6 million by 2050 [2]. Conventional treatments primarily manage the symptoms instead of targeting the underlying cellular mechanisms that drive the onset and progression of CVD. Thus, there is an urgent need for more effective strategies for the management of CVD, to improve the quality of life and reduce the burden on the healthcare system [3,4].

The hypothesis that oxidative stress contributes to CVD has gained traction ever since its first proposal by Daniel Steinberg and colleagues as a modifier of low-density lipoproteins (LDLs) [5]. In the mid to late 1990s, oxidative stress became a mainstream mechanism in CVD research; however, clinical trials using antioxidants like vitamin E failed to show a benefit [6,7,8]. These studies highlighted the complexity of redox regulation.

The term redox is derived from the combination of “reduction” and “oxidation,” which defines the chemical processes associated with the transfer of electrons between reactants in chemical reactions [9,10]. In the context of CVD, three classes of reactive species, namely, oxygen, nitrogen, and sulfur-based species, play central roles in redox regulation. Reactive oxygen species (ROS) are highly reactive molecular oxygen derivatives endogenously generated as a by-product of cellular respiration [11]. ROS, including superoxide (O_2_^•−^), singlet oxygen (^1^O_2_), hydrogen peroxide (H_2_O_2_) and hydroxyl radicals (^•^OH), are synthesized through redox processes [10,12]. H_2_O_2_ is recognized as a major ROS that reversibly oxidizes critical redox-sensitive cysteine residues on target proteins [11,13]. Various signal transduction pathways are regulated by ROS either via direct modification of proteins or lipids or by the coordinated transfer of electrons between molecules, forming a chain of redox reactions [14]. Redox signaling regulates numerous crucial physiological processes, such as insulin signaling [11], regulation of vascular tone [15] and immunometabolism [16].

Reactive nitrogen species (RNS) comprise nitrogen-containing radicals, including nitric oxide (NO) and nitrogen dioxide (NO_2_). NO acts as an important signaling molecule that regulates vasodilation, while excessive NO can react with superoxide to form the potent oxidizing and nitrating agent, peroxynitrite (ONOO^−^), which modifies protein tyrosine residues, causes lipid and DNA damage, alters protein function and dysregulates signaling pathways [17,18,19]. In doing so, RNS such as ONOO^−^ are associated with the pathogenesis of CVDs, including atherosclerosis, myocardial infarction (MI), hypertension and heart failure (HF) [19]. The generation of RNS varies across species, with evidence suggesting that human macrophages produce less RNS compared to mouse macrophages in response to inflammatory stimuli [20,21].

Reactive sulfur species (RSS), including hydrogen sulfide (H_2_S), sulfur dioxide (SO_2_), persulfides and polysulfides, have also gained attention as signaling molecules that are largely protective in CVD models, with a crucial role in regulating cardiovascular function in recent studies [22,23,24]. H_2_S also plays a pathophysiological role by interacting with nitric oxide, regulating post translational modification of proteins and modifying redox-dependent responses [23]. The H_2_S-induced post-translational modification, S-sulfhydrylation, has important roles in cellular processes, including cell metabolism, mitochondrial function, vasodilation, anti-inflammatory responses, redox imbalance and modifying endoplasmic reticulum stress in the cardiovascular system. In addition, SO_2_-induced S-sulfenylation is involved in maintaining vascular homeostasis [25]. Importantly, the H_2_S donor, S-propyl-L-cysteine, exerts cardioprotective effects by improving mitochondrial dysfunction via S-sulfhydration of Ca^2+^/calmodulin-dependent protein kinase II in heart failure [26]. However, dysregulated H_2_S signaling and its synthesizing enzymes can contribute to CVDs [22,23]. Although reactive nitrogen and sulfur species play significant physiological and pathophysiological roles, in this review, we primarily focus on ROS-mediated signaling in modulating inflammatory pathways, mitochondrial function, autophagy and CVDs.

Extensive investigations have shown that redox imbalance, mitochondrial dysfunction, impaired autophagy and unresolved inflammation are pivotal contributors to vascular and myocardial damage [27,28,29,30]. Dysregulated redox regulation facilitates persistent ROS generation and pathological feedback loops. Therefore, a comprehensive understanding of the contribution of redox signaling to cellular and molecular determinants of the various CVDs is critically needed for the development of novel therapeutic strategies.

Nonspecific antioxidants have not shown significant improvements in CVD outcomes in clinical trials [31,32]. These antioxidant approaches have largely failed due to a lack of specificity, inability to target the main sources of ROS and tendency to overlook the physiological roles of ROS in signaling and defense. Indeed, redox signaling is increasingly emerging as a pivotal player in metabolism and physiological processes, acting as a key mediator in the dynamic interactions between organisms and the external environment. A more nuanced approach to antioxidant interventions is needed, one that supports essential physiological redox processes yet affords protection against the onset and development of CVDs [10].

This review focuses on intracellular redox signaling and highlights the implications of redox imbalance in the pathophysiology of CVDs. Specifically, we focus on the root cause of redox imbalance, such as dysfunctional mitochondria, impaired endogenous antioxidant systems or impaired autophagy, and highlight their intricate interactions to discover novel therapeutic strategies. Furthermore, we highlight the complexity of redox signaling in CVDs, with a focus on the spatial and temporal aspects of ROS signaling. We highlight how ROS that originate from different cellular compartments, or temporal fluctuations in ROS, may have different effects on redox-sensitive transcription factors and organelle crosstalk. Non-canonical redox modifications, such as S-glutathionylation and S-nitrosylation, remain an active area of investigation and are discussed in relation to their role in modulating key pathways such as mitochondrial metabolism, autophagy and inflammation. Additionally, we address the bidirectional nature of redox interactions. Providing detailed insight into these aspects is essential for advancing our understanding of redox-mediated CVDs and developing effective mechanism-based therapeutic strategies.

## 2. Mechanisms of Intracellular ROS Generation

Mitochondrial complexes I (NADH: ubiquinone oxidoreductase) and III (ubiquinol: cytochrome c oxidoreductase) are considered the major sources of mitochondrial ROS (mtROS) produced by the electron transport chain [33]. These complexes, situated within the mitochondrial intermembrane space, generate O_2_^•−^ and H_2_O_2_ from molecular oxygen [33,34]. Mitochondria-localized proteins, such as NADPH oxidase-4 (NOX4), p66shc, monoamine oxidase (MAO)-A and MAO-B, have also been implicated in mtROS production [33]. In response to stress and p66Shc activation, cytochrome c in the inner mitochondrial membrane generates H_2_O_2_ [35], which exacerbates pro-apoptotic ROS signaling and mitochondrial dysfunction to drive a variety of cardiovascular pathologies [36]. While the primary sites and mechanisms of mitochondrial ROS generation are well characterized, their regulation and relative contributions to disease, and the physiological significance of each site, are not yet fully understood.

The regulation of ROS-mediated signaling is largely dependent on the spatiotemporal production of the ROS, which determines whether ROS exert physiological or pathological effects [9,11,37,38]. Indeed, the role of ROS is context-dependent and varies according to the cellular environment, compartmentalization, exposure period and concentration. In endothelial cells, oxidant radicals generated in one cellular organelle can affect ROS levels and function in other subcellular compartments [38]. It has been shown that communication between subcellular ROS, such as between mtROS and cytosolic ROS (cytoROS), substantially affects endothelial function and angiogenesis [38,39].

CytoROS play a crucial role in modulating numerous cellular signaling networks, whilst aberrant cytoROS disturb signaling pathways, thereby promoting pathophysiological changes [40]. The NOXs are a family of transmembrane enzymes involved in generating cytoROS. NADPH oxidase-2 was the first source of ROS identified in macrophages and is the canonical isoform in this cell type [34]. NOXs facilitate the generation of superoxide by transferring a single electron from NADPH to oxygen [41]. Superoxide can be further converted to H_2_O_2_ either through spontaneous dismutation or by the activity of superoxide dismutase (SOD) [34]. In addition, xanthine metabolism, specifically through the enzyme xanthine oxidase (XO), produces H_2_O_2_ and O_2_^•−^ in the cytoplasm.

Among the seven distinct isoforms of NOX, NOX-1, 2, 4 and 5 are expressed throughout the cardiovascular system [40,42,43]. Emerging data have revealed the crucial role of NOX2-derived O_2_^•−^ as signaling molecules in autophagy [40,44]. Notably, a study reported that NOX2-derived ROS present in LC3-associated phagosomes promoted oxidative inactivation of the autophagic protease ATG4B, thereby regulating its stability and function [44]. Similarly, in palmitate-treated H9c2 cardiomyocytes and in the hearts of mice fed high-fat-diets, activation of NOX2 enhanced O_2_^•−^ production, contributing to the inhibition of lysosomal enzymes and autophagosome turnover [42], suggesting that NOX-derived ROS play an important role in redox-dependent regulation of autophagy. Thus, modulating NOX activity and redox signaling to promote autophagy may offer a therapeutic avenue to restore cellular homeostasis and combat pathological remodeling of the heart.

Other sources of ROS include the endoplasmic reticulum (ER), peroxisomes and enzymes such as xanthine oxidase. The ‘redox triangle,’ formed by mitochondria, peroxisomes and the ER, acts as a central hub for redox signaling [45]. Excessive ROS within the redox triangle affects ER-mitochondria Ca^2+^ exchange, oxidative phosphorylation and protein folding within the ER [45]. In the human myocardium, the mitochondrial electron transport chain, NOX, xanthine oxidoreductase (XOR) and dysfunctional nitric oxide synthases (NOSs) are the major sources of ROS [46]. In the vasculature, ROS are generated by all the major vascular layers, including the endothelium, vascular smooth muscle cells (VSMCs) and adventitia [46]. Inflammatory cells are also major drivers of ROS production. Excessive ROS generated by both cardiovascular cells and infiltrating inflammatory and immune cells, such as neutrophils and macrophages, exacerbate inflammation and contribute to endothelial dysfunction, tissue damage and development of CVDs.

## 3. Cellular Antioxidant System

The antioxidant system is a highly coordinated defense network that provides protection from oxidative damage caused by ROS and other free radicals. Enzymatic and non-enzymatic antioxidants work synergistically to maintain the redox balance and cellular component integrity by modulating gene expression and associated signaling pathways. Thus, antioxidant therapeutics could provide an effective approach to preventing and treating many diseases where redox imbalance is a key pathological component, such as atherosclerosis, hypertension, ischemia–reperfusion injury and diabetic cardiomyopathy (DCM) [47,48]. The major enzymatic antioxidants include SOD, catalase (CAT) and the glutathione peroxidases (GPx), with glutathione reductase (GR), peroxiredoxins (Prx) and the thioredoxins (Trx) also maintaining the balance between oxidants and antioxidants. SOD catalyzes the dismutation of O_2_^•−^ into H_2_O_2_ and molecular oxygen, while catalase converts H_2_O_2_ into water and oxygen. In turn, GPx reduces hydrogen peroxide and lipid peroxides using glutathione as a substrate [49]. The thioredoxin–peroxiredoxin system detoxifies H_2_O_2_ and organic hydroperoxides by transferring reducing equivalents from NADPH, via thioredoxin reductase (TrxR) and thioredoxin, to Prx. The TrxR-Prx system also counteracts cysteine modifications via a reversible process that modifies oxidized cysteine thiol groups back to their reduced state, allowing proteins to function as redox-switches [50]. Of particular significance, excessive ROS can result in irreversible cysteine oxidation, leading to impaired protein function and disease progression [21]. The non-enzymatic antioxidants such as glutathione (GSH) and vitamins C and E actively scavenge free radicals and help regenerate oxidized antioxidants back to their active forms [49,51].

**Figure 1:**
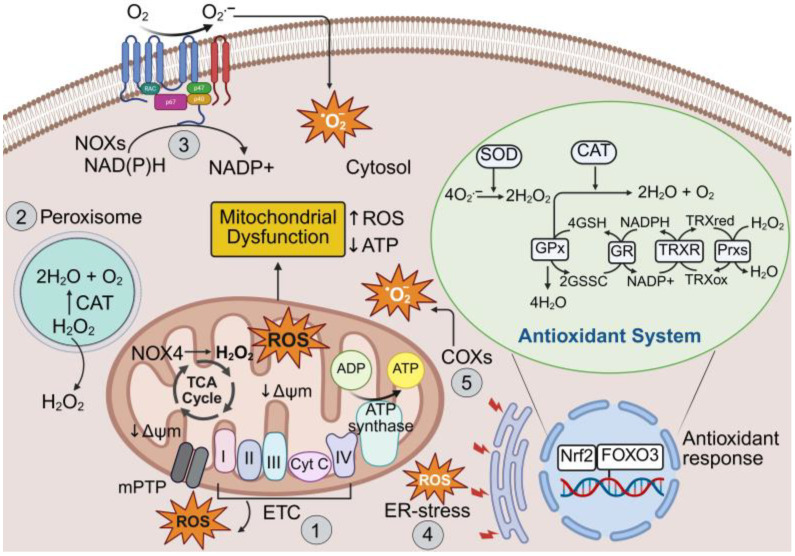
Intracellular reactive oxygen species (ROS) production and the cellular antioxidant defense system. ROS are produced by different cellular sources, primarily by (1) the mitochondrial electron transport chain (ETC), (2) peroxisomes and (3) NADPH oxidase (NOX). Other sources of ROS are the (4) endoplasmic reticulum (ER) and (5) cyclooxygenases (COXs). Mitochondrial O_2_^•−^ is produced by the electron transfer system via mitochondrial complexes I (NADH: ubiquinone oxidoreductase) and III (ubiquinol: cytochrome c oxidoreductase). NOX4, located in the mitochondrial membrane, generates H_2_O_2_, which leads to a decrease in the mitochondrial membrane potential (↓ΔΨm) and ultimately results in mitochondrial dysfunction. H_2_O_2_ is also produced in the peroxisomes via β-oxidation and is eliminated by catalase (CAT). The transcription factors, Nrf2 and FOXO3, orchestrate the cellular antioxidant response via the upregulation of antioxidant genes such as superoxide dismutase (SOD) and CAT, and enzymes involved in glutathione (GSH) synthesis. Enzymatic antioxidants (e.g., SOD, CAT and GPx) and non-enzymatic antioxidants (e.g., GSH) maintain the redox balance and cellular integrity by modifying gene expression and associated signaling cascades. Created in BioRender. Pervin, M.; de Haan, J.B. (2025) https://BioRender.com.

The transcription factor, Nrf2 (nuclear factor erythroid 2–related factor 2), acts as a central player and important redox switch in the regulation of the antioxidant system [52]. Upon activation by oxidative stress, Nrf2 translocates to the nucleus and binds to antioxidant response elements (AREs) of a range of genes, enhancing the expression of numerous cytoprotective enzymes such as SOD, CAT, Prx, heme oxygenase-1 (HO-1) and heat shock protein 70 (Hsp70) [17,53]. Together, these antioxidant defense systems help prevent oxidative stress-induced damage to DNA, proteins and lipids and modulate redox-sensitive signaling pathways involved in cell survival, inflammation and metabolism. The production of excessive ROS and the cellular antioxidant defense system required for cellular homeostasis is illustrated in Figure 1. Disruptions to this system lead to oxidative stress, contributing to the pathogenesis of various diseases, including CVDs.

## 4. Physiological Role of ROS, Redox Signaling and Redox Homeostasis

Redox signaling involves the specific and usually reversible oxidation/reduction modification of molecules involved in cellular signaling pathways [10,54], consequently turning on or off various pathways [55]. At low to moderate levels, ROS such as O_2_^•−^ and H_2_O_2_ contribute to normal cellular functions including proliferation, differentiation, migration and immune responses.

A key mechanism of redox signaling is the modification of cysteine residues in target proteins [21]. Oxidative post-translational modification of cysteine residues, such as S-nitrosylation, S-glutathionylation and sulfenylation, acts as redox switches that can modify the cellular response to oxidative stimuli. These modifications also alter the structure and function of sensor proteins that serve as mediators in redox signaling [24,37,56]. S-nitrosylation occurs upon the covalent attachment of NO to the thiol group (-SNO), whereas in S-glutathionylation reactions, the cysteine thiol forms a disulfide bond between the cysteine thiol and reduced glutathione [21]. These thiol-based post-translational modifications fine-tune mitochondrial function, autophagy and redox signaling, consequently regulating pathological cardiac remodeling and the onset and development of CVDs.

Moderately increased levels of mitochondrial oxidants enhance systemic defenses by inducing adaptive responses [57]. This is referred to as mitohormesis, the process where mitochondria signal in response to transient stress and activate adaptive cellular responses that increase cell survival, function and longevity [31,57,58]. Mitohormesis is increasingly viewed as an important aspect of normal physiology and a critical modulator of disease processes [58].

Physiological levels of H_2_O_2_ are in the range of 10 to 100 nanomolar (nM) [59]. H_2_O_2_ serves as a classical intracellular signaling molecule, modulating kinase-driven pathways at lower physiological levels [60]. The physiological steady-state levels of H_2_O_2_ are controlled by balancing H_2_O_2_ production and scavenging by antioxidant enzymes such as CAT and GPx [13]. Understanding the physiological function of ROS, and the importance of maintaining redox homeostasis, is critical for distinguishing beneficial signaling from pathological oxidative stress in the context of CVDs.

The antioxidant system scavenges excess ROS and ensures that redox signaling remains within the physiological range. Redox homeostasis refers to the precise balance between the generation of ROS and antioxidant activity. When this balance is regulated, redox signaling sustains the normal function of cells and tissues. However, excessive ROS production or impaired antioxidant responses lead to persistent redox imbalance, resulting in oxidative stress. This perturbs cellular homoeostasis, damages biomolecules, such as proteins, lipids and DNA, and leads to the pathogenesis of various diseases, including CVDs.

## 5. Dysregulated Redox Regulation: A Molecular Link to Inflammatory Pathways and Cell Death

**Figure 2:**
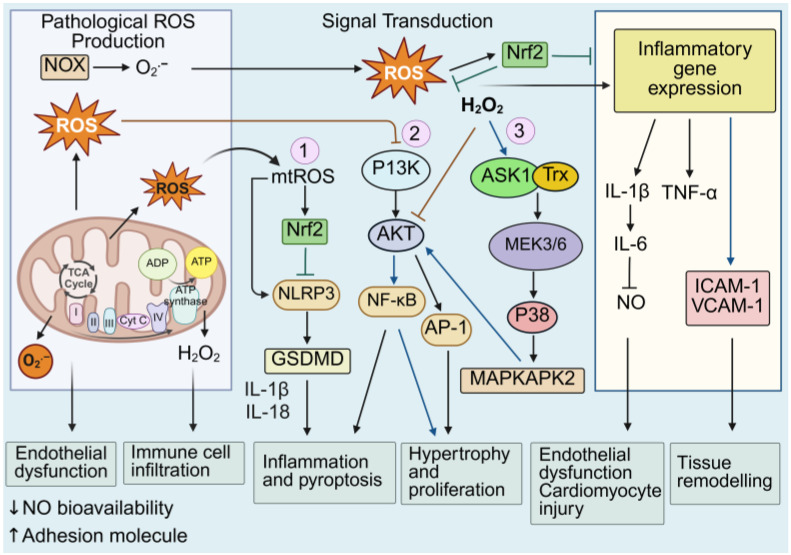
Mechanistic overview of ROS generation, redox signaling and pathological signal transduction leading to tissue damage and cell death in cardiovascular diseases. Mitochondria function as the major site of ROS production and as a central ROS-mediated signaling hub. ROS play an important role as second messengers in modulating multiple signaling pathways to regulate inflammation, including (1) components of the NLRP3 inflammasome, (2) PI3K-AKT-NF-κB and (3) MAPK pathways. In doing so, ROS activate the expression of inflammatory genes, including IL-1β, IL-6, TNF-α, VCAM-1 and ICAM-1, to enhance inflammatory and immune responses, ultimately accelerating the activation and progression of CVDs. Conversely, oxidative activation of Nrf2 inhibits inflammation by crosstalk with the NLRP3-inflammasome to lessen CVD. Created in BioRender. Pervin, M.; de Haan, J.B. (2025) https://BioRender.com.

Dysregulated redox regulation refers to disruptions in the intricate network of redox reactions within a biological system, resulting in persistent oxidative stress and altered redox signaling [9,10]. In the context of cardiovascular diseases, disruptions in redox homeostasis drive inflammatory and immune responses that accelerate the activation and progression of CVDs. Broadly speaking, redox-mediated processes include the dysregulation of the endothelium, enhanced pyroptosis (a novel form of programmed cell death) and inflammation, immune cell infiltration, cardiomyocyte hypertrophy and cellular proliferation, leading to tissue remodeling, which ultimately contributes to cardiovascular dysfunction and disease progression (Figure 2) [61,62,63]. More specifically, dysregulated redox signaling contributes to defective mitochondrial and autophagy pathways that amplify inflammatory signaling cascades such as the mitogen-activated protein kinases (MAPKs), nuclear factor kappa B (NF-κB) and the nucleotide-binding domain, the leucine-rich-containing family and the pyrin domain-containing-3 (NLRP3) inflammasome, whilst suppressing cytoprotective mechanisms including Nrf2-mediated antioxidant gene expression [40,64,65,66]. Multiple signaling cascades are activated or suppressed by interconnected redox-inflammatory regulators.

Evidence for the interconnectedness between redox regulation and inflammation comes from various in vitro and in vivo studies including the following. Immunological signaling, including via the Toll-like receptor (TLR) and NLRP3 inflammasome assembly, has been shown to require transient ROS generation before initiation of downstream signaling pathways [67,68]. Furthermore, ROS have been identified as regulators of inflammasome assembly. For example, inhibition of mitophagy leads to the accumulation of damaged and impaired mitochondria, which exacerbates ROS generation, and consequently triggers NLRP3 inflammasome activation [69,70]. In addition, the NLRP3-inflammasome is known to be activated by damage-associated molecular patterns (DAMPs) including oxidized DNA, lipids and proteins. ROS can directly oxidize mtDNA, targeting either the nucleobase or the sugar–phosphate backbone, and can also modify nucleotide bases prior to their incorporation during DNA polymerization [71,72]. Indeed, elevated ROS levels in cardiomyocytes and endothelial cells cause irreversible mitochondrial DNA damage, which impairs oxidative phosphorylation and mitophagy. Consequently, mtDNA and mitochondrial proteins are released into the cytosol and act as inflammatory stimuli that, via activation of the NLRP3-inflammasome, exacerbate tissue damage [72].

ROS also mediate the oxidative modification of LDL to generate the known atherogenic DAMP, oxidized low-density lipoprotein (oxLDL) [73]. OxLDL activates endothelial cells by promoting the expression of adhesion molecules on the cell surface [74]. These adhesion molecules facilitate leukocyte rolling and adhesion, and the migration of leukocytes into the intima layer in response to chemokines. This consequently triggers macrophage activation, pro-inflammatory cytokine secretion and the production of ROS and proteolytic enzymes, which contribute to matrix degradation, vascular inflammation and destabilization of plaques [73,75]. NLRP3 inflammasome activation leads to the production of interleukin-1β (IL-1β), which further induces the secretion of interleukin-6 (IL-6) and is implicated in the chronic inflammation and progression of CVD [76,77]. A recent study reported that IL-6 facilitates mtROS production and reduces NO bioavailability in human aortic endothelial cells, contributing to the development of endothelial dysfunction [78].

Growth factor stimulation has been shown to activate PI3K signaling via a redox-sensitive mechanism [63]. Tu et al. demonstrated that oxidative stress activates PI3K and increases the activity of p70 S6 kinase-1, leading to enlargement of cardiomyocytes [79]. In VSMCs, the ROS-sensitive kinase, p38 MAPK, and its substrate, MAPKAPK-2, have been shown to mediate AKT activation, which contributes to VSMC hypertrophy [80].

Data from our laboratory demonstrated that dh404, a bardoxolone derivative and novel Nrf2 activator, ameliorates endothelial dysfunction in diabetic Akita mice by activating Nrf2, upregulating antioxidant enzymes, reducing ROS and inhibiting redox-sensitive inflammatory pathways. In diabetic human aortic endothelial cells (HAECs), dh404 showed cytoprotective effects by significantly inhibiting inflammatory genes (VCAM-1 and the p65 subunit of NF-κB) and upregulating the Nrf2-responsive genes, NAD(P)H quinone oxidoreductase 1 (NQO1) and HO-1, whilst decreasing the oxidative stress marker, nitrotyrosine, and the ROS, O_2_^•−^ and H_2_O_2_. In diabetic mice, dh404 decreased contraction in response to phenylephrine and suppressed the expression of inflammatory genes, including VCAM-1, ICAM-1, p65 and IL-1β, as well as pro-oxidant genes, Nox1 and Nox2 [66]. We also showed that dh404 reduces inflammation and atherosclerosis in diabetic apolipoprotein E knockout (*Apo^−/−^*) mice [81]. Our data therefore highlight the interconnectedness between dysregulated redox pathways and inflammation and suggest that specific, targeted antioxidant therapy may lessen CVD burden via improvements in oxidative stress and inflammation.

ROS play a major role in regulating all forms of programmed cell death, including pathways that regulate apoptotic and non-apoptotic cell death such as necroptosis, pyroptosis and ferroptosis, leading to the death of cardiomyocytes and adverse cardiac remodeling [82,83,84]. For example, the fusion protein, PEP-1-MsrA, has been shown to inhibit H_2_O_2_-induced oxidation and to suppress apoptosis and necroptosis in macrophages. Methionine sulfoxide reductase A (MsrA) inhibited atherogenesis by reducing the intracellular ROS level, inflammatory responses and apoptosis in atherosclerotic lesions in Western diet-fed *ApoE^−/−^* mice [85]. ROS have been found to induce vascular endothelial cell apoptosis and to promote the progression of atherosclerosis. In support of this, kansuinine, a macrocyclic diterpenoid isolated from Euphorbia kansui, suppressed H_2_O_2_-induced intracellular ROS generation in HAECs and inhibited apoptosis via the IKKβ/IκBα/NF-κB pathway [86]. In *ApoE^−/−^* mice, kansuinine treatment downregulated apoptosis-related protein, elevated the level of GPx and malondialdehyde (MDA) and significantly reduced atherosclerotic lesions. Ferroptosis has been shown to play an important role in the pathogenesis of cardiac ischemia–reperfusion injury and diabetic cardiomyopathy [87,88]. Specifically, in streptozotocin (STZ)-induced T2D mice, Wang et al. demonstrated that ferroptosis is a major player in the development of diabetic cardiomyopathy, while the Nrf2 activator, sulforaphane, prevented cardiac ferroptosis and associated pathogenesis via AMPK-mediated Nrf2 activation [88]. However, distinct ROS species elicit specific cell death responses dependent on the ROS type and subcellular localization.

In summary, accumulating evidence suggest that cellular redox imbalance plays a crucial role in driving a cascade of redox-sensitive signaling events and inflammatory pathways. This exacerbates cellular and tissue damage, ultimately leading to the development and progression of various CVDs. Advancing our understanding of how disrupted redox signaling exacerbates inflammatory and immune responses may assist in the discovery of novel therapeutic approaches to restore redox balance and regulate inflammation-associated pathologies.

## 6. The Crosstalk Between Redox Signaling and Mitochondrial Function

Mitochondria are double membrane-bound organelles that are known to generate most of the energy needed to power biochemical reactions of the cell [89]. In 1966, Jensen initially reported that the mitochondrial respiratory chain generates ROS [90,91]. It was later established that H_2_O_2_ is produced from the dismutation of O_2_^•−^ in the mitochondria [91,92,93]. Mitochondria constitute approximately 30–40% of the cardiomyocyte cell volume and play a crucial role in meeting the high metabolic and energy demand by primarily generating ATP through oxidative phosphorylation (OXPHOS) [94]. A growing body of literature now supports the notion that mitochondria are both a major source and the target of ROS, positioning them at the center of vital redox signaling networks. The bidirectional interaction between mitochondrial function and redox homeostasis forms a complex axis that regulates energy production, survival, and stress responses. Disruptions of the interplay between mitochondrial function and redox homeostasis may contribute to the pathogenesis of numerous diseases, including cardiovascular and metabolic disorders [89,95].

Mitochondrial dysfunction leads to the dysregulation of mitochondrial dynamics, mitochondrial DNA (mtDNA) damage, and impaired mitophagy [89]. Dysfunctional mitochondria also contribute to inflammation and an impaired immune response [96]. Dysfunctional mitochondria affect calcium homeostasis and cardiac energy supply, which causes changes in cardiac structure and function [89]. Therefore, dysfunctional mitochondria are associated with many cardiovascular diseases, including atherosclerosis, heart failure, and myocardial infarction [30,89,97,98].

Accumulating evidence suggests that mtROS function as downstream effector molecules. mtROS can modulate various signaling pathways such as modulation of hypoxic signaling [99,100], cytosolic stress kinases [101] and activation of autophagy [102], thereby influencing cell metabolism and immune responses. In particular, mitochondrial oxidative stress has been shown to directly impact the inhibitor kappa-B kinase β (IKKβ)–RelA (NF-κB) pathway. Indeed, mitochondrial oxidative stress leads to increased monocyte infiltration and exacerbated inflammatory responses in Western-diet-fed Ldlr^−/−^ mice. Conversely, decreasing mitochondrial stress in macrophages alleviated atherosclerosis by reducing monocyte infiltration and lesional inflammation in an mCAT transgenic (mCAT) Ldlr^−/−^ mouse model of atherosclerosis [64]. Furthermore, NF-κB-induced oxidative stress contributed to mitochondrial and cardiac dysfunction in obese db/db mice, a model of type II diabetes. Notably, inhibition of NF-κB by the NF-κB inhibitor, pyrrolidine dithiocarbamate, reduced oxidative stress, restored mitochondrial integrity, and increased ATP synthesis, consequently improving cardiac function [103].

mtROS also crosstalk with the NLRP3 inflammasome to drive inflammatory responses. In addition to a direct effect on the activation of the NLRP3 inflammasome [104], a recent study demonstrated that cardiomyocyte-specific knockdown of a protein involved in autophagic flux, ATP6AP2, led to autophagy inhibition and activation of the NLRP3 inflammasome, further promoting maladaptive cardiac remodeling. In contrast, suppression of cellular and mitochondrial ROS in shR-ATP6AP2-transfected cardiomyocytes partially reversed NLRP3 upregulation and mitigated mitochondrial impairment and dysfunction [102]. Thus, cellular and mitochondrial ROS promote activation of the NLRP3 inflammasome, which may contribute to cardiac dysfunction.

mtROS also act as upstream signals that promote Nrf2 activation by disrupting its interaction with KEAP1, thereby facilitating its nuclear translocation and transcriptional activation of antioxidant genes. A recent study by Luo et al. demonstrated that in oxLDL-injured macrophages, micheliolide (MCL), an active metabolite of parthenolide, reduced both total and mtROS levels, increased SOD activity, improved mitochondrial function, modulated antioxidant responses and, importantly, reduced atherosclerosis. Mechanistically, MCL binds to the Arg483 site of KEAP1, enhancing Nrf2 nuclear translocation and upregulating the transcription of GPx4 and xCT. These findings suggest that MCL ameliorates atherosclerosis by activating the Nrf2 signaling pathway and thereby reducing oxidative stress and the inflammatory response [105]. Furthermore, mtROS play a bidirectional role in regulating mitochondrial dynamics via modulation of mitochondrial fission and fusion, while these processes also influence mtROS production [40].

Excessive ROS can enhance mitochondrial fission by activating the major pro-fission protein dynamin-related protein 1 (DRP1) [89]. ROS-induced post-translational modifications such as phosphorylation, SUMOylation, S-nitrosylation, and O-GlcNAcylation play an important role in DRP1 activation [106,107]. Cytosolic DRP1 is recruited to mitochondrial membranes following post-translational modifications and interacts with the outer mitochondrial membrane protein Fis1 to initiate mitochondrial fission [106]. Additionally, three crucial GTPase proteins, Mitofusins 1 (MFN1) and Mitofusins 2 (MFN2) on the outer membrane and atrophy 1 (OPA1) on the inner membrane, mediate mitochondrial fusion [89]. Oxidative stress can inhibit mitochondrial fusion by impairing the function of key fusion proteins. In H9c2 cardiomyoblasts, H_2_O_2_-mediated oxidative stress disrupts OPA1-mediated mitochondrial dynamics via activation of OMA1, a key protease responsible for cleavage of OPA1, implicating a crucial role of ROS in mitochondrial dynamics [108]. Inhibition of mitochondrial fission promotes the accumulation of dysfunctional mitochondria, which further exacerbate ROS generation. Similarly, impaired mitochondrial fusion in endothelial cells enhances superoxide production, which leads to atherosclerosis progression [109], highlighting the complex bidirectional link of ROS and mitochondrial dynamics and function.

In addition, mtROS play a role in mediating lytic cell death via oxidation of the pore-forming protein, gasdermin D (GSDMD), thereby promoting pyroptosis of macrophages [110,111]. The Regulator-Rag complex, a mediator of mTOR activity, is involved in GSDMD pore formation and pyroptosis in macrophages [112]. The Regulator–Rag complex regulates mTORC1-dependent events to promote oligomerization of GSDMD and pore formation in the membrane via a mtROS-mediated process. However, the exact mechanism by which mtROS affects GSDMD oligomerization is not clearly understood [112]. Redox regulation of proteins can be mediated by direct modification of thiol-containing amino acid residues such as cysteines [10]. Devant et al. demonstrated that ROS enhances GSDMD activities through oxidative modification of multiple cysteine residues, with cysteine 192 (Cys192) being necessary and sufficient for ROS-mediated GSDMD pore formation and pyroptosis [111]. Thus, mtROS can activate the NLRP3-dependent pyroptosis pathway by inducing the oxidation of GSDMD, which damages cardiomyocytes and myocardial tissue, leading to various cardiovascular conditions, including cardiac hypertrophy, atherosclerosis, and myocardial reperfusion injury [113,114,115].

Furthermore, recent studies report that redox regulation plays a key role in modulating other components of the NLRP3-GSDMD axis, including NLRP3, apoptosis-associated speck-like protein containing a CARD (ASC), cysteinyl aspartate-specific proteinase-1 (Caspase-1), and NIMA-related kinase 7 (NEK7) [116,117,118]. For example, small molecules such as imiquimod and CL097 inhibit the quinone oxidoreductases, particularly NQO2, and mitochondrial Complex I, resulting in excessive ROS generation and thiol oxidation, which facilitates NEK-mediated NLRP3 activation [116]. Indeed, deglutathionylation of NEK7, at cysteine 253, has been shown to promote NLRP3 inflammasome activation [119]. Additionally, most of the NLRP3 agonists, such as ATP, nigericin, and cholesterol crystals, exacerbate ROS production and promote inflammasome assembly and activation, while conversely, the transcription factor Nrf2 inhibits NLRP3 activation by reducing oxidative stress [68,118,120]. Also, mtROS have been shown to activate the NLRP3 inflammasome through K^+^-independent NLRP3 activation [116]. Taken together, most evidence suggests that ROS can either trigger or inhibit NLRP3 inflammasome activation depending on the source, localization, concentration and context. Importantly, restricting ROS-mediated activation of the NLRP3-GSDMD axis under pathological conditions that promote CVD appears critical for preserving cellular homeostasis and preventing tissue damage.

Furthermore, oxidative post-translational modification (Ox-PTM) of mitochondrial proteins can modulate ATP synthesis, electron transport efficiency and calcium handling [56,121]. For example, in the failing heart, ATP synthase undergoes oxidative modification at multiple cysteine residues via disulfide bond formation, S-glutathionylation and S-nitrosation. It has been shown that Cys294 of the ATP synthase α subunit acts as a redox switch that senses the cellular redox status and modulates ATP synthase activity [121]. Importantly, cardiac resynchronization therapy has been shown to restore ATP synthase function, partially by reversing oxidative modifications on cysteine residues [121].

Redox signaling can also influence the mitochondrial structure and function by regulating Ca^2+^ flux. Mitochondrial Ca^2+^ homeostasis is primarily maintained by Ca^2+^ influx into the matrix via the mitochondrial calcium uniporter (MCU), whilst the main efflux process is mediated by the Na^+^/Ca^2+^ exchanger (NCX) [122,123]. Recent studies demonstrated that redox modification of MICU3 regulates mitochondrial calcium influx [10,95,124]. Patron et al. reported that the novel tissue-specific MCU modulator, MICU3, forms a disulfide bond with MICU1 at the Cys515 residue, which stimulates mitochondrial Ca^2+^ uptake [10,124]. Another study found that oxidation of MCU at cysteine 97 (Cys-97) also increased MCU activity. Cysteine 97 is a conserved thiol residue in human MCU, and it has been shown to undergo S-glutathionylation, thereby increasing MCU activity [123,125]. This oxidative modification of MCU further enhances mtROS production, disrupts cellular bioenergetics and sensitizes cells to mitochondrial calcium [Ca^2+^]_m_ overload-induced cell death [125]. Thus, redox modifications directly regulate Ca^2+^ homeostasis, which can impact the development and progression of CVDs. Therefore, exploring the underlying mechanisms by which dysfunctional mitochondria contribute to oxidative imbalance within the cell, and how the redox-sensitive targets modulate mitochondrial function, may provide critical insights to discover more effective therapeutic targets for CVDs.

## 7. The Crosstalk Between Redox Signaling and Autophagy

The autophagy–lysosome system is a highly conserved cellular process that degrades damaged cellular content and maintains homeostasis [126,127]. The autophagy–lysosomal process is involved in three main types of autophagy: microautophagy, chaperone-mediated autophagy and macroautophagy. These processes provide the cell with a flexible degradative toolkit for different conditions. Microautophagy degrades cytoplasmic material through direct lysosomal membrane invaginations, while chaperone-mediated autophagy selectively transports proteins bearing a KFERQ motif across the lysosomal membrane via LAMP-2A [128,129]. Macroautophagy, commonly referred to as autophagy, involves the engulfment of cytoplasmic components within double-membrane vesicles called autophagosomes, which then fuse with the lysosome, allowing enzymatic degradation and recycling of the sequestered material [127,130]. The autophagy process is mediated by an intricate interplay of multiple proteins and lipids derived from various membrane sources, including the endoplasmic reticulum, ER/mitochondria contact sites, the Golgi apparatus, recycling endosomes and the cell membrane [127,131]. More than 32 related proteins are associated with the autophagosome before fusion to the lysosome [132]. Mechanistic target of rapamycin (mTOR) and AMP-activated protein kinase (AMPK) are important regulators of autophagy [133,134]. Downstream, proteins such as Beclin-1, part of the PI3K complex, drive phagophore nucleation, the process that initiates the formation of the autophagosome. Microtubule-associated protein 1A/1B-light chain 3 (LC3) is converted to LC3-II through a lipidation process driven by the action of ATG7 and ATG3. LC3-II anchors to the autophagosomal membrane, allowing it to facilitate cargo recruitment and enhance autophagosome formation [135]. These autophagosomes subsequently fuse with lysosomes, which allow lysosomal enzymes to recycle the sequestered material. Unc51-like autophagy activating kinase 1 (ULK1) acts as a central player in the autophagy initiation process by forming a complex with other proteins [131]. mTOR can suppress ULK1 complex activity by binding and phosphorylating ULK1 at Ser757 and ATG13 at Ser258 [136]. In a recent study, Tabata et al. revealed the mechanism by which the ULK1 complex targets autophagosome formation and regulates autophagy initiation. They found that zinc finger DHHC-type palmitoyltransferase 13 (ZDHHC13) palmitoylates ULK1 during autophagy induction and enhances downstream events such as phosphorylation of ATG14L [131]. Notably, palmitoylation of ULK1 occurs specifically at cysteine residues, Cys927 and Cys1003 [131].

**Figure 3:**
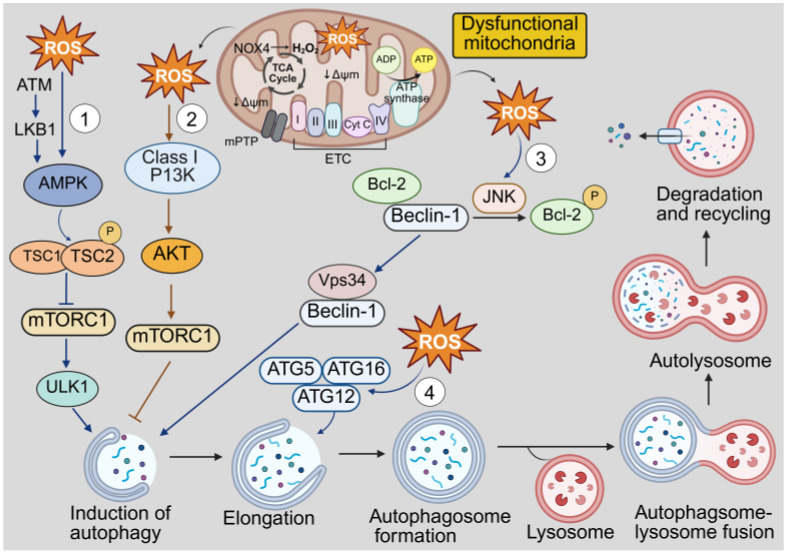
Redox regulation of autophagy. The complex autophagy process initiates with the formation of a phagophore, followed by its elongation, autophagosome formation, fusion with the lysosome to form an autolysosome, and degradation and recycling of cellular components. ROS regulate autophagy in a context-dependent manner and can either promote or inhibit this process. Distinct mechanisms are denoted by numbered circles. (1) Excessive ROS trigger the activation of AMPK, which phosphorylates TSC2. This activates the tuberous sclerosis complex 1/2 (TSC1/TSC2), leading to the inhibition of mTORC1 and subsequent activation of ULK1, which promotes autophagy initiation and progression. ROS also directly oxidize and activate AMPK; (2) ROS-mediated activation of the PI3K -AKT pathway promotes mTORC1 activity, which in turn inhibits autophagy initiation, and depending on the cellular and pathophysiological context, ROS induce autophagy through activation of the PI3K/AKT/mTORC1 signaling pathway; (3) ROS activate Beclin1-dependent autophagy, and during oxidative stress, activated JNK directly phosphorylates Bcl-2 and enhances the dissociation of Bcl-2 from Beclin 1, which allows Beclin1 to interact with the Vps34 complex to activate autophagy; (4) ROS promote the activation of the ATG12-ATG5 complex, which facilitates autophagy activation. ATM: ataxia-telangiectasia mutated, LKB1: liver kinase B1. The black arrows indicate the process of autophagy. The brown arrows indicate the PI3K-AKT pathway. The blue arrows indicate the AMPK-mTOR and ROS-JNK-mediated autophagy pathways. Created in BioRender. Pervin, M.; de Haan, J.B. (2025) https://BioRender.com.

Under normal physiological conditions, macroautophagy plays a crucial role in cell survival and homeostasis. However, dysfunctional autophagy is associated with many diseases, including cardiovascular and metabolic diseases [137,138,139]. Autophagy can be activated by oxidative stress, amino acid starvation, reduced insulin levels and reduced ATP availability [140]. Importantly, ROS regulate autophagy in a complex and context-dependent manner to either activate or suppress autophagy via multiple signaling pathways including activation of AMPK signaling and the ULK1 complex, inhibition of Bcl-2/Beclin-1 and mTOR signaling and direct oxidation of ATG proteins [130,141,142,143,144] (Figure 3). The redox-mediated regulation of autophagy facilitates cellular adaptations to stress and plays a key role in the inflammatory response, metabolic balance and cardiovascular pathophysiology [145,146].

AMPK is a highly conserved master regulator of metabolism, and it plays a central role in regulating autophagy, particularly under oxidative and energy stress [130,147,148]. ROS activate AMPK through multiple mechanisms, leading to suppression of mTORC1 and autophagy induction [130]. Excessive ROS activate ataxia-telangiectasia mutated (ATM), which promotes activation of TSC2 tumor suppressor through the LKB1/AMPK pathway, leading to suppression of mTORC1, ultimately facilitating autophagy induction [130,149]. Excessive ROS typically induce autophagy through inhibition of mTORC1. However, under certain conditions, ROS enhance mTORC1 activity and subsequently inhibit autophagy. Elevated ROS can directly oxidize and activate AMPK [130]. Moreover, ROS have been shown to both activate and suppress PI3K/AKT/mTORC1 signaling by complex and context-dependent mechanisms, which are not completely understood [130,150]. A traditional Chinese medicinal formulation, Shengjie Tongyu, exerts a protective effect against diabetic myocardial injury by modulating the ROS-PI3K/AKT/mTOR axis by LncRNA H19, thereby inhibiting autophagy in cardiomyocytes [150]. Another study reported that hydroxysafflor yellow A-sonodynamic therapy induces autophagy through ROS-derived activation of the PI3K/AKT/mTOR signaling pathway, consequently reducing inflammation in THP-1 macrophages [151]. Furthermore, in response to oxidative stress, JNK activation leads to phosphorylation of Bcl-2, consequently dissociating Beclin1 from the Vps34 complex and activating autophagy [142,144]. It has also been demonstrated that ROS trigger the induction of autophagy by activating a ubiquitin-like protein complex, Atg12-Atg5 [130,144]. Moreover, direct thiol oxidation of key regulatory proteins such as ATG4, ATM (serine/threonine kinase) and transcription factor EB (TFEB) fine-tunes autophagy flux by selectively regulating phagophore elongation, cargo recognition and transcription of autophagy genes, respectively [152].

Additionally, excessive ROS can activate multiple important transcription factors such as hypoxia-inducible factor-1α (HIF-1α), Nrf2, p53 and forkhead box O-3 (FoxO3), which can activate the transcription of autophagy-related genes including SQSTM1, LC3 and the mitophagy-associated genes BNIP3 and NIX [153,154]. In addition to activating upstream signaling pathways, redox stress can also directly modify key autophagy-related proteins such as ATG4, Beclin-1 and p62/SQSTM1 via oxidative post-translational modifications, consequently affecting the efficiency and specificity of the autophagic process. For example, ATG4, a cysteine protease that processes LC3, is reversibly inhibited by ROS, acting as a redox-sensitive switch to regulate formation of the autophagosome [152,155]. Redox modifications, particularly oxidation of the autophagy receptor p62, can also affect its oligomerization and cargo recognition ability, impacting selective autophagy [156]. Carrol et al. found that two oxidation-sensitive cysteine residues, C105 and C113, in the autophagy receptor SQSTM1/p62, facilitate the activation of pro-survival autophagy under stress [156]. These reversible redox modifications facilitate fine-tuning of the autophagic machinery in response to the changing redox state of the cell, thereby modulating autophagy. In addition to this, autophagy has been found to indirectly regulate ROS by p62-mediated selective degradation of Keap1, which results in the release and activation of Nrf2, and upregulation of antioxidant target genes, thereby reducing ROS levels [130,142]. A classic activator of Nrf2, tBHQ, has been found to attenuate oxidative stress and suppress VSMC calcification by inducing Nrf2 nuclear translocation and increasing P62 and KEAP1 expression [157].

Autophagy, in turn, maintains redox homeostasis by eliminating ROS-producing dysfunctional mitochondria (via mitophagy) and degrading oxidized proteins and lipids, thereby maintaining mitochondrial functional integrity and cellular homeostasis [158,159]. This intricate interplay of autophagy and redox signaling limits oxidative damage in cells and tissues. A better understanding of the mechanism of autophagy in various diseases is crucial for therapeutic target design and the treatment of diseases [159]. Notably, ROS have been found to activate PINK1-Parkin-mediated mitophagy by inducing mitochondrial recruitment of Parkin [159,160]. SIRT3 also plays a key role in activating PINK1/Parkin-mediated mitophagy, by deacetylating PINK1 and Parkin directly or through the transcription factor FOXO3a [161]. Furthermore, the production of localized mtROS during metabolic stress or hypoxia serves as a source of key upstream signaling molecules for the induction of mitophagy [162]. However, under certain conditions, mtROS are also elevated as a result of the induction of mitophagy [162].

From the aforementioned studies, it is clear that redox-dependent autophagic regulation is crucial for the adaptation to cellular stressors to maintain the energy balance and quality control of proteins and organelles. In the cardiovascular system, cardiomyocytes and vascular endothelial cells have high metabolic demands and are frequently exposed to oxidative stress. Therefore, a more detailed mechanistic understanding of redox-sensitive checkpoints within the autophagic pathway, particularly in specific pathological contexts, could lead to the development of novel therapies for CVDs.

## 8. Interconnected Signaling and Feedback Loops: The Redox-Mitochondria–Autophagy–Inflammation Axis

The complex and bidirectional relationship between redox signaling, mitochondrial dysfunction, autophagy, mitophagy and inflammation is gaining attention particularly in the context of cardiovascular and metabolic diseases. However, the interconnectedness of these processes in relation to immunometabolic regulation and the onset and progression of CVDs is not completely understood. These interconnected signaling pathways create complex feedback loops that drive the progression and development of different cardiovascular conditions.

Research has shown that ROS function as important secondary messengers that activate transcription factors such as NF-ĸB and AP-1, promoting the secretion of pro-inflammatory cytokines such as IL-1β, IL-6 and TNF-α. These cytokines, in turn, trigger more ROS production by activating the NOX family of enzymes and disrupting mitochondrial electron transport, thereby facilitating a positive feedback loop between redox imbalance and inflammation. Similarly, increased ROS levels contribute to mitochondrial dysfunction, while impaired mitochondrial activity is associated with excessive ROS generation [163]. In particular, excess ROS disrupt mitochondrial integrity by damaging mitochondrial membranes, DNA and proteins, whereas impaired mitochondria become the main sources of further ROS production, triggering cellular stress and promoting damage to cardiomyocytes [163,164]. Furthermore, impaired autophagy is commonly observed in aging and metabolic syndromes where damaged mitochondria accumulate, leading to persistent ROS production and chronic inflammation [165,166].

One of the major players regulating the cellular stress response is sirtuin1 (SIRT1), a class III histone deacetylase. SIRT1 is considered a crucial regulator of oxidative stress and has been shown to play a role in modulating CVDs, including atherosclerosis, myocardial infarction and heart failure [167,168,169]. SIRT1 mitigates inflammation by deacetylating NF-ĸB, p53 and PGC-1α during metabolic perturbations [170,171,172]. In oleic acid-treated VSMCs, SIRT1 deacetylases PGC-1α, restores mitochondrial dysfunction and improves mitochondrial membrane potential [173]. SIRT1 promotes mitochondrial biogenesis, enhances the autophagy process and reduces oxidative stress, and thus it plays a crucial role in modulating redox-mediated cellular processes.

A further key regulator of this process is p62/SQSTM1. Multiple studies have demonstrated that p62/SQSTM1 sits at the intricate nexus of redox signaling, mitochondrial quality control, autophagy and the inflammatory response [174,175,176,177]. However, it plays a context-dependent role in cellular homeostasis, acting both as a marker of impaired autophagy and as a mediator of protective responses. Specifically, p62 is an autophagic receptor/adaptor protein that shuttles damaged cargo into the autophagosome, but its accumulation reflects impaired autophagic flux and is typically associated with adverse cellular outcomes. With respect to complications of CVD, a recent study revealed that p62/SQSTM1 regulates oxidative and ER stress, and inflammation following cerebral I/R injury, with elevated p62 levels being associated with worse stroke outcomes. Mechanistically, the ZZ domain of p62 was shown to mediate dysregulated autophagy and cell death through the binding of specific substrates, especially those containing an N-terminal arginine (Nt-R). This interaction initiated p62 oligomerization, and subsequent autophagosome formation, and yet the degradation of cargo was dysregulated [178]. In addition, Quan et al. found that p62 increased mitochondrial ROS in a NOX-independent manner in HEK293T cells after an I/R exposure. Importantly, accumulation of p62 under impaired autophagy conditions leads to prolonged inflammatory activation [179]. This underscores its importance as a therapeutic target in inflammation-driven cardiovascular diseases.

p62 has also been shown to mediate mitophagy by binding to ubiquitinated outer mitochondrial membrane proteins and recruiting autophagic machinery, thus degrading damaged and dysfunctional mitochondria and limiting the production of mitochondrial ROS [159,180]. Another study demonstrated that SQSTM1/p62 positively regulates mtDNA expression and mitochondrial OXPHOS [176]. In addition, SQSTM1/p62 induced the expression of mitochondrial ribosomal protein MRPL12 by activating the p38/ATF2 signaling pathway, suggesting a new regulatory axis [176]. Thus, given its context-dependent roles in regulating autophagy, oxidative stress responses and inflammation, targeting p62 may offer a novel approach for CVD prevention; however, therapeutic strategies will need to carefully balance its protective functions with potential risks associated with p62 accumulation and impaired autophagic flux.

P66Shc, a key adapter protein, is yet another key regulator of oxidative stress with an impact on inflammatory outcomes. P66Shc controls the progression of various cardiac pathologies, including endothelial dysfunction, coronary artery disease (CAD), ischemia/reperfusion injuries and cardiomyopathy [36]. P66Shc has been shown to regulate cardiac dysfunction and oxidative stress in a mouse model of pressure overload-induced heart failure (TAC model), with SOD and phosphodiesterase 5 (PDE5) acting as downstream effectors of this pathway [181]. P66Shc is phosphorylated under oxidative stress and translocates to the mitochondria, where it enhances H_2_O_2_ generation and promotes mitochondrial permeability transition, thereby contributing to oxidative damage, apoptotic signaling, exacerbation of the inflammatory cascade and cellular dysfunction [182,183,184].

Importantly, these studies show the complex crosstalk between the pathways regulating redox signaling, mitochondrial homeostasis, autophagy and inflammation, and how crosstalk may amplify pathological signaling. This challenges therapeutic targeting, since targeting one pathway may inadvertently affect others. However, by identifying and modulating key regulatory hubs within this network, such as Nrf2, the NOX enzymes or mitochondrial quality control proteins, novel therapeutic strategies could restore multiple dysfunctional pathways, leading to broader cardioprotection.

## 9. Interplay of Autophagy, Mitochondrial Dysfunction and Cellular Redox States in the Context of CVDs

**Figure 4:**
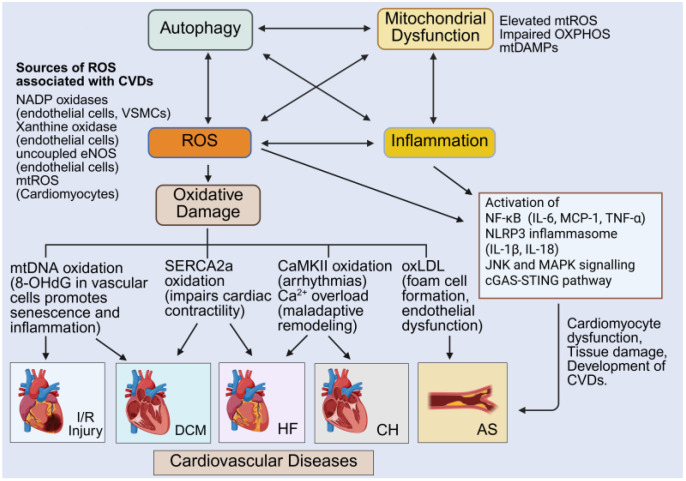
Interconnectivity of the redox–mitochondria–autophagy–inflammation axis in the pathophysiology of CVDs. This leads to increased oxidative damage and inflammation, affecting downstream inflammatory mediators, and SERCA2a, CaMKII, oxLDL and mtDNA, which ultimately contribute to the progression of CVDs, including ischemia/reperfusion (I/R) injury, diabetic cardiomyopathy (DCM), heart failure (HF), cardiac hypertrophy (CH) and atherosclerosis (AS). Created in BioRender. Pervin, M.; de Haan, J.B. (2025) https://BioRender.com.

The intricate crosstalk between autophagy, mitochondrial dysfunction, ROS production and elevated inflammation is now increasingly being recognized as central to CVD progression (Figure 4). Clinically, these processes underpin key features of cardiovascular pathology, including endothelial dysfunction, cardiomyocyte death and adverse cardiac remodeling. The following section highlights the direct involvement of these interconnected pathways in the pathogenesis of atherosclerosis, cardiac hypertrophy, ischemia/reperfusion injury, heart failure and diabetic cardiomyopathy, and how an understanding of their interconnectivity might inform newer therapeutics to lessen the disease burden.

### 9.1. Atherosclerosis

Atherosclerosis is a chronic immunometabolic disease that develops at multiple locations within the arterial system, and I is the primary cause of CVD, including coronary artery disease (CAD), stroke, myocardial infarction (MI) and peripheral artery disease. It is also a major contributor to heart failure, especially heart failure with a reduced ejection fraction (HFrEF) after MI. At a cellular level, endothelial cells, VSMCs and leukocytes contribute to the development and progression of atherosclerotic lesions [185,186]. In response to a pro-atherogenic environment, an inflamed vascular endothelium attracts monocytes into the subendothelial space of the vessel wall. It is within this space that monocytes internalize oxLDL particles via scavenger receptors such as CD36, scavenger receptor-1 (SR-1) and lectin-like oxidized LDL receptor (LOX-1) to cause a build-up of plaque in the arterial wall [187].

Initial mechanistic investigations highlighted a critical role for ROS in the development of atherosclerosis. This led to the proposal of the oxidative modification hypothesis of atherosclerosis in 1989 [5]. This theory proposed that oxLDL is a key driver of macrophage-derived foam cell formation within the arterial wall. Subsequent studies proved that ROS function as mediators of multiple processes of atherosclerosis progression, including endothelial dysfunction, foam cell formation and plaque destabilization [188]. In the ensuing years, chronic inflammation was acknowledged as an additional key mediator [189], where an imbalance between pro-inflammatory and anti-inflammatory processes ultimately drives the onset and development of atherosclerotic plaques [190]. Furthermore, the importance of mtROS in mediating atherogenic processes is considered a nuanced advance in our understanding of ROS-mediated atherogenesis [191,192].

Over a decade ago, Wang et al. reported that mitochondrial oxidative stress is associated with the development of human atherosclerosis [64]. The crosstalk between mitochondrial dysfunction, ROS and inflammation in driving atherosclerosis was highlighted in their study by showing that mitochondrial oxidative stress in macrophages enhanced monocyte chemotactic protein-1 (MCP-1) production via the IKKβ–RelA NF-κB pathway. Notably, suppression of mitochondrial oxidative stress in myeloid cells inhibited early atherogenesis in Ldlr^−/−^ mice overexpressing catalase in their mitochondria [64]. However, how cytoplasmic IKK was activated by mitochondrial oxidative stress remains unclear. More recent studies continue to support the interconnectedness of these pathways and draw attention to the importance of NOX4-driven mitochondrial ROS in pro-inflammatory macrophage reprogramming. For example, Vendrov et al. show that ablation or pharmacological inhibition of NOX4 reduced mitochondrial ROS, skewed macrophages towards a resolving M2-like phenotype and attenuated plaque progression in *ApoE*^−/−^ mice [193].

The aforementioned observations highlight how mtROS signaling acts as a crucial regulator of macrophage polarization. Activated macrophages are classically categorized into two distinct phenotypes: M1 and M2 macrophages [194]. M1 macrophages play a crucial role in initiating and sustaining inflammatory responses, whereas M2 macrophages are involved in tissue repair, wound healing and anti-inflammatory responses [37]. In reality, macrophages exist in a continuum between the M1 and M2 extremes, with intermediate subsets present under normal physiological conditions to maintain immune homeostasis. A sustained shift away from this balanced state is often associated with disease progression [195]. In this regard, ROS play a key role in regulating the functional polarization of macrophages and, consequently, cellular homeostasis. Studies demonstrate that mtROS act as key signaling molecules to drive macrophage reprogramming towards the M1-like phenotype [195,196]. Multiple studies demonstrate that modulating macrophage polarization from a pro-inflammatory M1-like state to an anti-inflammatory M2 phenotype may protect against plaque instability and rupture, suggesting that M1/M2 macrophage polarization-targeted treatment may provide a new approach for atherosclerosis treatment [197,198,199].

Given the pivotal role of redox status in dictating macrophage polarization and function, strategies to restore mitochondrial integrity and enhance autophagy have gained attention. Evidence for this notion comes from data of Karnewar et al., who demonstrated that a mitochondria-targeted antioxidant, esculetin (Mito-Esc), significantly prevented dysregulation of mitochondrial biogenesis in the aortas of *ApoE^−/−^* mice whilst reducing serum pro-inflammatory cytokines and preventing atherosclerotic plaque formation. Furthermore, in human aortic endothelial cells and serum from *ApoE^−/−^* mice, Mito-Esc activated the metabolic and stress-sensing autophagy regulator, SIRT1, altered miR-19b and miR-30c and significantly inhibited plasminogen activator inhibitor-1 (PAI-1), a key mediator of atherosclerosis [200]. These data suggest that via targeted reductions in mitochondria-mediated oxidative stress, it is possible to improve mitochondrial dysfunction and augment autophagy to reduce inflammation and cellular damage in CVDs.

Importantly, the interconnectedness of the autophagy and oxidative stress pathways is revealing novel therapeutic opportunities for atherosclerosis therapy. In a study by Xia et al., the significance of targeting AMPK/mTOR-dependent autophagy in atherosclerosis was shown in *ApoE*^−/−^ mice [201]. In these mice, inhibition of autophagy by U0126 resulted in an increase in aortic atherosclerosis with increased necrotic core and foam cell formation. Mechanistically, P62 was shown to accumulate together with a decrease in lactoferrin (LTF), an iron transport protein with anti-inflammatory, antioxidant and antifibrotic properties, mainly secreted by neutrophils. A decrease in autophagosomes was also noted, suggesting that autophagy was impaired. Using in vitro cell models, the study also showed that silencing the core autophagy protein, BECN1, or knocking down LTF increased mTOR phosphorylation, inhibited the expression of LC3 II and prevented the activation of AMPK, all indications that autophagy was impaired. This study suggests that dysregulated autophagy and high levels of oxidative stress are associated with the development of atherosclerosis, and that lactoferrin therapy might ameliorate atherosclerosis by accelerating the AMPK/mTOR signaling pathway [201]. Thus, advances in our understanding of the complex role of oxidized lipids and redox signaling can open avenues for novel therapeutic interventions for the treatment of atherosclerosis and aid in the development of strategies to prevent or slow plaque development.

For example, in light of their known antioxidative, anti-inflammatory and vasoprotective properties, renewed interest in high-density lipoproteins (HDLs) has focused on strategies aimed at enhancing HDL functionality rather than simply increasing circulating HDL-cholesterol levels [202]. HDL plays an essential role in reverse cholesterol transport, a process that carries cholesterol from peripheral tissue to the liver for excretion [203]. Moreover, HDL can inhibit LDL oxidation and neutralize pro-inflammatory lipids, thereby modulating immune cell activation within atherosclerotic lesions. These properties provided a compelling rationale for HDL-based therapies. However, clinical trials aimed at raising plasma HDL-cholesterol levels such as those using niacin, or early CETP inhibitors, largely failed to reduce cardiovascular events [204,205]. This has redirected attention toward improving HDL functionality, including its cholesterol efflux capacity and anti-inflammatory activity, rather than simply increasing HDL-C levels. Current therapeutic strategies now focus on short-term infusion of reconstituted HDL or apoA-I mimetics (e.g., CSL112) to enhance cholesterol efflux and stabilize vulnerable plaques in the acute post-myocardial infarction setting [206,207], and on next-generation CETP inhibitors such as obicetrapib [202] that more favorably modulate lipid profiles. Although these approaches have shown mixed results to date, recent large-scale trials have renewed optimism that restoring HDL function may still offer a clinical benefit in selected contexts.

### 9.2. Pathological Cardiac Hypertrophy

Pathological cardiac hypertrophy arises due to pressure overload, hypertension or aortic stenosis, and it is an independent risk factor for cardiovascular diseases. It is a hallmark of heart failure and sudden death, and it is typically characterized by an increase in cardiomyocyte size and left ventricular wall thickening [208,209]. A number of studies suggest that multiple signaling mediators contribute to the development of pathological cardiac hypertrophy by disrupting normal cellular functions, including mitochondrial respiration, calcium handling, metabolic regulation and autophagy. This manifests as alterations in oxidative stress and inflammation [209,210]. Indeed, ROS have been shown to play a crucial role in regulating multiple overlapping signaling pathways associated with the development and progression of pathological cardiac hypertrophy [210]. In particular, the role of mtROS and the interplay with a ROS modulator are clearly demonstrated in a recent study by Martens et al. Reactive oxygen species modulator 1 (ROMO1) is an inner mitochondrial membrane protein that influences mitochondrial dynamics and redox signaling. It facilitates ion flux via ion channel formation and affects mitochondrial membrane potential to drive ROS production. It is highly expressed in hypertrophic hearts resulting from transverse aortic constriction (TAC) surgery, and overexpressing ROMO1 is associated with developing hypertrophy in human AC16 cardiomyocytes. Notably, knockdown of ROMO1 markedly reduces ROS production and inhibition of NF-κB activity, suggesting the ROMO1-ROS-NF-κB signaling axis is involved in the regulation of pathological cardiomyocyte hypertrophy [211]. This study clearly highlights the interconnectivity between oxidative and inflammatory stress in pathological hypertrophy, and it suggests that redox signaling acts as a central mediator in the development of cardiac hypertrophy. Advancing our knowledge of the critical role of redox signaling in hypertrophy may reveal potential therapeutic targets to prevent maladaptive remodeling and heart failure.

Although a role for autophagy has been implicated in pathological cardiac hypertrophy, conflicting data, as demonstrated below, suggest both a positive and a negative effect of autophagy on disease progression. A recent study demonstrated that solute carrier family 26 member 4 (SLC26A4), also known as pendrin, promotes autophagy and activation of the NLRP3 inflammasome in two cardiac hypertrophy models, the first a mouse model of phenylephrine (PE)-induced cardiomyocyte hypertrophy, and the second a rat model of transverse aortic constriction (TAC) [212]. The mechanism most likely involves its anion exchange activity that influences cellular stress pathways to promote the development of cardiac hypertrophy. In isolated cardiomyocytes, protein levels of NLRP3 and IL-β were downregulated after treatment with the autophagy inhibitor 3-MA, or after silencing with a sh-lentivirus expressing SLC26A4. These data suggest that SLC26A4 mediates the activation of both autophagy and the NLRP3 inflammasome to promote the progression of cardiac hypertrophy both in vitro and in vivo.

On the other hand, a natural compound, thymoquinone, decreased the levels of key hypertrophic markers, ANP and BNP, and reduced type 1 collagen expression in angiotensin II (AngII)-treated H9c2 cells and TAC mice, consequently mitigating cardiac hypertrophy. Importantly, the mechanism included activating adaptive autophagy through the PPAR-γ/14-3-3γ pathway [213]. Additionally, thymoquinone markedly decreased the level of ROS by upregulating NOX4 and SOD2 in both angiotensin II (AngII)-treated H9c2 cells and TAC mice, indicating a crucial role for autophagy and oxidative stress in pathological cardiac hypertrophy [213]. Taken together, these studies reveal that the role of autophagy is highly context-dependent, varying with the cellular environment; therefore targeting impaired autophagy and aberrant inflammasome activation may provide new therapeutic strategies for pathological cardiac hypertrophy. Furthermore, mitochondrial impairment is one of the major drivers of pathological cardiac hypertrophy [211,214,215]. In AngII-treated rat cardiomyocytes, overexpression of the resident mitochondrial protein, SBK3, reduced the level of mtROS and malonaldehyde, a marker of oxidative stress, in cardiomyocytes by increasing SOD2 activity. In addition, SBK3 overexpression restored the expression of mitochondrial dynamics-related proteins, including MFN1 and MFN2. Concurrently, SBK3 overexpression increased ATP production, improved the respiratory and oxygen consumption rate of cardiomyocytes and, consequently, improved cardiac hypertrophy by regulating mitochondrial metabolism [215]. In addition, downregulation of the cardiac-specific mitochondrial fission-regulating protein, Drp-1, promotes the accumulation of damaged and dysfunctional mitochondria and consequently increases oxidative stress in the heart during pressure overload-induced cardiac hypertrophy, whereas Tat-Beclin 1 peptide treatment activates mitophagy and restores mitochondrial function, thereby alleviating the progression of HF during pressure overload [216]. These data highlight the complex role of mitochondrial dysfunction, autophagy and oxidative stress in cardiac hypertrophy and the progression of heart failure.

### 9.3. Ischemia–Reperfusion (I/R) Injury

Ischemia–reperfusion (I/R) injury results in cardiac damage and dysfunction, which elevates the risk of heart failure. This occurs when blood flow to the heart is interrupted, resulting in excessive mitochondrial ROS generation upon reperfusion, largely due to the accumulation of TCA cycle intermediates, such as succinate, driving HIF-1α-mediated ROS production [11]. Myocardial cell death induced by myocardial I/R plays a central role in the progression of acute myocardial infarction (AMI), mainly via necrosis, apoptosis and autophagic death [217,218]. Myocardial I/R injury expands the infarct area, contributes to the aggregation of inflammatory cells in the ischemic myocardium, impairs vascular endothelial function and causes metabolic dysfunction and apoptosis of myocardial cells, all of which exacerbate AMI [218,219].

Multiple studies underscore the protective role of autophagy in the cardiac response to ischemia via removal of damaged mitochondria and the reduction in oxidative stress [220,221]. A study demonstrated that I/R injury of the rat heart promotes the accumulation of ROS and metabolic dysfunction of the mitochondria. In this study, mitochondrial sequesteration by the autophagasome was reduced in I/R rat hearts compared to control hearts, whilst this was improved by corosolic acid treatment. Mechanistically, it could be shown that corosolic acid exerted its protective effects by enhancing mitophagy through the PHB2/PINK1/Parkin signaling pathway, which facilitated the elimination of damaged mitochondria, decreased oxidative stress and maintained mitochondrial function, consequently reducing the infarct size and improving cardiac function post-I/R injury in rats [220].

RhoA, a small G-coupled protein receptor and intracellular signal transducer, has been implicated in cardioprotective mechanisms post-I/R injury. Activation of RhoA signaling reduces oxidative stress via suppression of mitochondrial death pathways [222,223,224]. Tu et al. demonstrated that activation of RhoA upregulates exogenously expressed PINK1 and Parkin within the mitochondria. RhoA activation increased the level of LC3-II in mitochondria, and this increase remained unaffected by Bafilomycin A1 treatment, indicating that RhoA promotes the induction of mitophagy rather than affecting lysosomal degradation. This sustains mitochondrial quality control by modulating mitophagy [224]. Thus, PINK1-mediated mitophagy contributes to the clearance of impaired mitochondria and safeguards cardiomyocytes from ischemic injury [224]. Another recent example of protection afforded by enhanced mitophagy comes from data investigating the protective effects of two xanthone derivatives isolated from Garcinia bracteata, Gerontoxanthone I (GeX1) and macluraxanthone (McX), that promote the activation of mitophagy through the PINK1-Parkin pathway and reduce the levels of ROS. Consequently, these compounds were shown to reduce injury and cell death of H9c2 cardiomyoblasts [225]. Collectively, these studies highlight the protection afforded by mitophagy against oxidative stress and I/R injury.

In contrast, other studies report that excessive or dysregulated mitophagy may exacerbate injury [226,227,228]. For example, during simulated ischemia reperfusion (SIR) in H9c2 monocytes, mitophagy was highly activated, which exacerbated oxidative stress and mitochondrial dysfunction. Treatment with melatonin decreased the levels of mitophagy-associated proteins, including Beclin1, Parkin, Bcl-2/adenovirus E1B 19-kDa-interacting protein 3 (BNIP3) and NIX (BNIP3-like (BNIP3L), reduced the levels of ROS and restored mitochondrial function by reducing mitochondrial permeability transition pore (mPTP) opening and suppressing cyclophilin D (CypD) and voltage-dependent anion channel 1 (VDAC1) expression in H9c2 cells. These results suggest that melatonin protects H9c2 cells from SIR-induced injury by inhibiting excessive mitophagy [228]. These differential outcomes may reflect differences in the severity of the insult, cell type or regulatory pathways involved in mitophagy activation, suggesting that mitophagy plays a context-dependent role in I/R injury.

In addition, there is evidence for a role for autophagy in mediating I/R injury. Depletion of the transcription factor ZNF143 has been shown to improve autophagic flux in myocardial I/R injury and decrease cardiomyocyte death, whereas overexpression of ZNF143 upregulates Raptor expression and inhibits autophagic activity, consequently exacerbating myocardial I/R injury. These data suggest that the regulation of impaired autophagic flux attenuates myocardial I/R injury [229]. Conversely, another study demonstrated that the DEP-domain-containing mTOR-interacting protein (Deptor) ameliorates I/R-induced myocardial injury by inhibiting the mTOR pathway and by increasing cardiomyocyte autophagy [218]. Furthermore, in a setting of diabetes, disrupted autophagic flux leads to augmented I/R injury in STZ-induced hyperglycemic mice [230], whilst I/R injury is aggravated in patients with diabetes [231,232].

The rate of ROS generation rapidly increases in the post-ischemic myocardium [233]. ROS generated from multiple sources have been implicated in ischemia–reperfusion injury. Mitochondria-localized circular RNAs (circRNAs), a newly identified class of non-coding RNAs, play an important role in regulating the production of mitochondria-derived ROS in cardiomyocytes [234]. Mitochondria-localized circRNA Samd4 decreases oxidative stress and regulates mitochondrial dynamics by inducing mitochondrial translocation of valosin-containing protein (Vcp), consequently decreasing voltage-dependent anion channel 1 (Vdac1) expression and inhibiting mPTP opening [234]. These results highlight how a mitochondrial non-coding circRNA regulates mitochondrial function and protects cells from oxidative stress, via a pathway that facilitates protein translocation into the mitochondria to alter gene expression and pore regulation. In addition, overexpression of circSamd4 induces cardiomyocyte proliferation and inhibits cardiomyocyte apoptosis, which results in improved cardiac function after AMI. By modulating circSamd4 or its downstream targets (Vcp or Vdac1), it may be possible to develop therapeutic strategies aimed at enhancing mitochondrial resilience and mitigating cellular damage [234].

Taken together, these studies provide strong evidence for the complex role of redox signaling, mitochondrial dysfunction and autophagy in the pathogenesis of AMI-induced cardiac injury, and they suggest that considerations of dose, timing, cell type and disease stage are needed for effective strategies to treat I/R injury.

### 9.4. Heart Failure

Heart failure (HF), also known as congestive heart failure (CHF), is defined as a complex clinical syndrome where the heart is unable to pump blood effectively due to structural or functional impairments in ventricular filling [235]. HF affects at least 26 million people worldwide and contributes to high mortality and morbidity, poor quality of life and increased healthcare costs. Increasing evidence suggest a close link between oxidative stress and heart failure [17,236]. In particular, ROS-mediated damage to cellular macromolecules, such as lipids, proteins and DNA, leads to cell death and loss of cardiac contractile function [17]. Importantly, electron leakage from dysfunctional mitochondria leads to the formation of superoxide radicals [237], thereby amplifying oxidative stress and contributing to the development of heart failure [238]. Growing evidence from both animal studies and clinical observations reinforces the notion that excessive mtROS significantly exacerbate cardiac pathology in the failing heart [238,239,240]. Notably, excessive mitochondrial oxidative stress may act both as a cause and as a consequence of mitochondrial dysfunction during the progression to heart failure [240].

Strong evidence supports the interconnected role of ROS, mitochondrial dysfunction and autophagy in heart failure. Indeed, it has been found that the dopamine D5 receptor (D5R) reduces the production of mtROS in a cAMP and autophagy-dependent manner [241]. Notably, cardiac-specific dopamine D5R knockout mice (Drd5 myh6fl/fl-creERT2) develop hypertrophic cardiomyopathy and heart failure via mechanisms that lead to increased NADPH oxidase activity and ROS production and mitochondrial dysfunction, whilst antioxidant administration (Apocynin, Tempol, Mito-TEMPO) rescued the cardiac hypertrophy and fibrosis [242]. Interestingly, myeloid differentiation protein 1 (MD1) has been shown to enhance the rate of cardiomyocyte autophagy in heart failure with a preserved ejection fraction (HFpEF) by activating the ROS-mediated MAPK signaling pathway [243].

Emerging evidence now highlights a complex interplay between the key regulators of the cellular stress response such as autophagy, hypoxic signaling and the regulation of oxidative stress, in driving cardiac fibrosis and the progression to heart failure [177,244,245]. In particular, a study by Ghosh et al. reported that the selective autophagy adaptor protein, p62, reduces hypoxia-induced cardiac dysfunction by stabilizing HIF-1α and Nrf2 [177]. In H9c2 rat cardiomyoblasts, depletion of p62 enhances proteasomal degradation of Nrf2, whereas overexpression of p62 stabilizes Nrf2 levels, suggesting a crucial role for p62 in HIF-1α and Nrf2 stabilization and transcriptional activity to maintain the redox balance and protect the cell from hypoxic stress [177].

Moreover, a high level of oxidative stress can cause myocardial fibrosis by enhancing the proliferation of cardiac fibroblasts and collagen production, consequently stiffening the heart muscle and impairing its contractile and diastolic function, ultimately leading to heart failure [17,246]. A recent study described a mechanism by which ROS facilitate the proliferation of cardiac fibroblasts [246]. In a mouse model of cardiac fibrosis induced by Ang II or ischemia–reperfusion injury, elevated levels of miRNAs containing oxidized guanosine (O8G) modifications were observed. It was shown that treatment with Ang II or PDGF induced excess ROS, which resulted in oxidative modification of guanosine (G) to 8-oxoguanosine (O^8^G) in miR-30c. Modified miR-30c downregulated CDKN2C, a negative regulator of cardiac fibroblast proliferation, thereby enhancing proliferation of fibroblasts and excessive accumulation of extracellular matrix [246].

Finally, a role for nitrosylation has been demonstrated in a preclinical model of heart failure. S-nitrosoglutathione reductase (GSNOR), located in the mitochondria of cardiomyocytes, has been shown to play a crucial role in regulating mitochondrial homeostasis via the denitrosylation of adenine nucleotide translocase 1 (ANT1) at cysteine 160. ANT1 controls mitochondrial energy exchange by facilitating ATP export and ADP import across the mitochondrial inner membrane. Indeed, an impaired GSNOR/ANT1 axis exacerbated heart failure by promoting mitochondrial dysfunction and dysregulated mitophagy [247]. In addition, oxidative modification of ATP synthase, H-Ras and histone deacetylase4 (HDAC4) has been shown to drive progression of cardiac remodeling leading to heart failure [24,121,248,249], underscoring the crucial role of oxidative thiol modifications in CVDs.

Collectively, redox imbalance, mitochondrial dysfunction and dysregulated autophagy contribute to a deleterious feedback loop that amplifies cardiac remodeling and accelerates the progressive loss of cardiac function, which is characteristic of heart failure.

### 9.5. Diabetic Cardiomyopathy

Diabetic cardiomyopathy (DCM) is characterized by an abnormal myocardial structure and function without the presence of additional cardiac risk factors such as coronary artery disease, hypertension and severe valve disease in diabetic patients [250]. DCM has emerged as the main cause of heart failure in diabetic individuals [251]. The underlying mechanisms of DCM are multifactorial and not yet fully understood. Oxidative stress is a key factor in the pathogenesis of DCM [252]. Numerous factors are implicated in the development of DCM, including impaired insulin and metabolic signaling, impaired glucose uptake, oxidative stress, mitochondrial dysfunction, autophagy, mitophagy, imbalance between matrix metalloproteinases (MMPs) and tissue inhibitors of metalloproteinases (TIMPs), impaired Ca^2+^ handling and inflammation [251,252].

Numerous preclinical and clinical studies have demonstrated the crucial role of oxidative stress in DCM [253,254,255,256]. Mechanistically, hyperglycemia accelerates mitochondrial electron transport chain activity, leading to electron leakage and excessive ROS generation, thereby driving oxidative stress [257]. In an experimental DCM model, metformin suppressed the expression of COL-I, III, TGF-β, CTGF, ICAM and VCAM genes, reduced collagen deposition and improved cardiac function by reversing DCM-associated damage [255]. Given its ability to activate AMPK, which in turn enhances mitochondrial efficiency and lessens mtROS, these effects are likely mediated by the known ability of metformin to mitigate oxidative stress [258]. Another study reported that in high-glucose-induced H9c2cells, naringenin inhibits ROS generation, reduces inflammatory cytokine production and suppresses apoptosis. Similarly, in type 1 diabetic mice, naringenin reduces oxidative stress and inflammation by inhibiting NF-κB and enhancing Nrf2 activity, consequently alleviating cardiac fibrosis and cardiomyocyte apoptosis [259]. With respect to diabetic patients, a clinical study reported high levels of mtROS and an increase in the inflammatory markers, NFκB-p65 and TNF-α, in T2D leukocytes, which correlated with increased inflammatory and vascular complications, whilst MitoQ treatment enhanced antioxidant defenses (GPx1 levels) in T2D leukocytes and lessened chronic inflammation and the risk of CVD [260]. Furthermore, a naturally occurring flavonoid, Kaempferol, alleviated hyperglycemia-induced cardiac injury and apoptosis by inhibiting oxidative stress and inflammatory responses specifically through inhibition of NF-κB nuclear translocation and activation of Nrf2 in in vitro studies and in diabetic mice hearts [256]. Finally, myricitrin (Myr), another naturally occurring flavonoid, has been shown to reduce ROS and inflammatory cytokines, leading to reduced apoptosis in advanced glycation end-product (AGE)-induced H9c2 cardiomyocytes [261]. Similarly, Myr treatment of streptozotocin-induced diabetic mice inhibited the production of inflammatory cytokines and apoptotic proteins, and it downregulated the expression of enzymes associated with cardiomyopathy, as well as improved diastolic dysfunction. Mechanistically, it could be shown that Myr alleviated oxidative stress and inflammation via the AKT-dependent activation of Nrf2 signaling whilst inhibiting the NF-κB pathway [261].

Emerging evidence suggests that targeted modulation of T cells offers a promising strategy to attenuate DCM. In a recent study, regulatory T cells were shown to reduce oxidative stress, inflammation and apoptosis, thereby attenuating myocardial hypertrophy and fibrosis and improving cardiac dysfunction [262]. T cells also protected against the progression of DCM in db/db mice by regulating the PI3K–AKT and MAPK signaling pathways [262].

Collectively, across atherosclerosis, pathological cardiac hypertrophy, ischemia–reperfusion injury, heart failure and diabetic cardiomyopathy, strong preclinical and clinical evidence supports the notion that the convergence of oxidative stress, mitochondrial dysfunction, impaired autophagy and chronic inflammation initiates amplifies and propagates CVDs, and that elucidation of the crosstalk relevant to each pathology may reveal mechanistically informed and specific targets for therapeutic intervention.

## 10. Refining Redox Approaches for CVD: From Vitamins to Precision Therapies

As discussed above, preclinical evidence strongly supports a role for redox imbalance as a critical mediator of cardiovascular diseases. The data suggest that redox signaling can be targeted for the prevention of CVDs and that modulating the cellular redox state through lifestyle, dietary and pharmacological interventions could be an important strategy to reduce the risk of onset and progression of CVDs, particularly in older adults who are more likely to develop symptoms. Unfortunately, clinical translation of antioxidant therapy with vitamins such as vitamin A or E has not proved efficacious in large-scale clinical trials such as HOPE (Heart Outcomes Prevention Evaluation), HOPE-TOO and GISSI-Prevenzione [263,264,265]. HOPE-TOO evaluated long-term supplementation of vitamin E (400 IU/day) in high-risk CV patients and found no reduction in major adverse CV events (MACEs); it was instead associated with an increased risk of heart failure [264]. Similarly, the GISSI trial, which evaluated post AMI patients, showed no benefit from vitamin E therapy in reducing CV death or other MACE outcomes [265]. More recently, a systematic review and meta-analysis of 38 studies showed that vitamin E did not prevent or reduce the mortality of CVDs in most trials [8]. Additionally, some studies reported that high-dose vitamin E may have detrimental effects on cardiovascular outcomes [8]. These studies question the translational validity of earlier preclinical findings. However, the failure of these trials may be multifactorial, namely, it is proposed that vitamin strategies often involve indiscriminate antioxidant activity; they may disrupt beneficial redox signaling and paradoxically, through their mechanism of action, vitamins generate further ROS. Vitamins may additionally fail to localize effectively to subcellular compartments where oxidative damage is most relevant. These limitations, along with the negative outcomes of the large-scale clinical trials, raise important questions about targeting ROS for CVD prevention, but importantly have highlighted that a more nuanced approach to antioxidant therapy is needed. Emerging evidence now suggests that pharmacologically targeting redox-sensitive pathways, such as the activation of Nrf2, the inhibition of NADPH oxidases or targeting of therapies to the mitochondria, may offer a more precise and physiologically attuned approach to restoring redox balance.

Indeed, there is strong evidence supporting a role for mitochondria-targeted antioxidants, such as MitoQ or SkQ1, in improving cardiovascular outcomes. Effective delivery of therapeutic compounds to the mitochondria in vivo is challenging [266]. MitoQ is a chemically modified version of CoQ10 with an added triphenylphosphonium cation (TPP^+^) to assist with its translocation and accumulation in the mitochondria [266,267]. MitoQ has been shown to restore age-related decreases in endothelium-dependent dilation (EDD), to reduce aortic stiffness and to improve vascular function in both old mice and clinical studies of older adults without adverse effects [268,269]. Furthermore, in a randomized, placebo-controlled, double-blind crossover study, MitoQ was found to improve endothelial function partially by reducing mtROS in middle-aged and older adults [269]. In-depth mechanistic insights into the mode of action of CoQ10 and MitoQ are covered in Section 11.3 of this review. With respect to Nrf2 activators such as bardoxylone methyl, clinical translation for CVDs has been limited by adverse CV events (most likely due to fluid overload), as seen in the BEACON trial [270]. NADPH oxidase inhibitors such as GKT1378312 have shown promise in reducing vascular oxidative stress and fibrosis in animal studies [43]; however, robust clinical data supporting their use in CVD prevention remain lacking.

## 11. Therapeutic Implications and Challenges

Considering the disconnect between preclinical and clinical data with respect to antioxidant therapies, future strategies need to address the type of antioxidant, the source of ROS production, the duration of treatment, the dosing regimen and the population-specific requirements to avoid off-target effects, including modulation of physiological redox signaling essential for cellular homeostasis. A promising strategy might be to focus on preventing the production of reactive oxidants that damage cellular macromolecules such as DNA, proteins and lipids by targeting key enzymatic sources such as NADPH oxidases, xanthine oxidase and dysfunctional mitochondrial complexes. Moreover, inhibiting downstream redox-sensitive signaling pathways, such as NF-ĸB, MAPKs and the NLRP3 inflammasome that drive inflammation, fibrosis and programmed cell death, should be the focus of newer antioxidant and anti-inflammatory strategies. Augmenting endogenous antioxidants by enhancing endogenous antioxidant enzymes, including SOD, catalase and GPx, as well as modulating redox[sensitive transcription factors, such as Nrf2, could provide protection against redox imbalance-associated diseases. Additionally, identifying specific small molecules or drug targets that improve mitochondrial dynamics, biogenesis and mitophagy processes may facilitate the development of targeted therapeutic interventions for CVDs.

Therapeutic modulation of autophagy depends on the disease context, since the role of autophagy is complex and context-dependent. Thus, autophagy can be therapeutically targeted using agents such as AMPK activators, mTOR inhibitors or TFEB inducers to promote cellular clearance and stress adaptation. Similarly, when excessive autophagy contributes to cell damage, lysosomal blockers or autophagy initiation can restore or modulate impaired autophagy. The current understanding of these themes is explored below.

### 11.1. Targeting the Oxidative Stress–Mitochondria–Autophagy–Inflammation Axis

**Figure 5:**
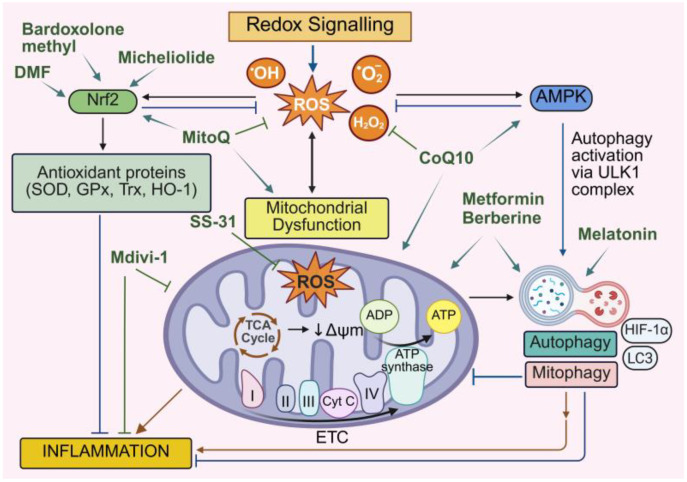
Targeting the redox–mitochondria–autophagy–inflammation axis: novel strategies for the treatment of CVDs. Activating or inhibiting redox-mediated signaling pathways provides a unique opportunity to develop novel therapeutic targets for the prevention and treatment of various CVDs. MitoQ and CoQ10 neutralize ROS such as superoxide (O_2_^•−^) and the hydroxyl radical (^•^OH) to prevent pathological redox signaling. Small molecules such as micheliolide, bardoxolone methyl and dimethyl fumarate (DMF) bolster Nrf2 levels to drive Nrf2-mediated responses that include upregulated antioxidant defenses (superoxide dismutase (SOD), glutathione peroxide (GPx), thioredoxin (Trx) and heme-oxygenase-1 (HO1)). Additionally, metformin, berberine and melatonin stimulate autophagy via activation of AMPK, ULK1, HIF-1α and LC3 to lessen inflammatory responses. SS-31 inhibits mitochondrial ROS production, thereby improving mitochondrial biogenesis, whilst Mdivi-1 blocks both inflammation and mitochondrial dysfunction. These are some of the newer strategies to lessen oxidative stress and inflammation in the quest for more targeted approaches to lessen CVD. Created in BioRender. Pervin, M.; de Haan, J.B. (2025) https://BioRender.com.

The integrated network of interactions that form the redox signaling–mitochondria–autophagy–inflammation axis has significant implications for the pathogenesis of CVDs. Targeting these pathways may prevent cardiovascular inflammation and tissue damage by suppressing oxidative stress and regulating mitochondrial dynamics and complex autophagic flux (Figure 5).

### 11.2. Targeting Oxidative Stress in Cardiovascular Disease

Numerous preclinical studies have demonstrated the protective role of the major antioxidant enzymes against oxidative stress and tissue damage in mouse models of cardiovascular disease [271,272,273,274]. For example, a deficiency of CuZnSOD enzyme activity in *Sod1* KO mice augmented Nox2 levels in the heart and led to oxidative damage and dysfunctional cardiac function [273]. Extracellular superoxide dismutase (EC-SOD) has been shown to ameliorate hypoxia-induced epigenetic modifications of the tumor suppressor gene, RASSF1A, by modulating the Ras/ERK pathway and decreasing fibrosis and tissue damage [273]. The clinically approved SOD mimic and redox-active drug, MnTnBuOE-2-PyP^5+^ (BMX-001), has been shown to inhibit human valve interstitial cell activation and extracellular matrix remodeling, in a murine model of aortic valve sclerosis [275].

Our lab and others have shown a protective role for GPx1 in the prevention of atherosclerosis [276]. We demonstrated a significant increase in plaque burden in diabetic *ApoE/GPx1* double knockout (dKO) mice, which was accompanied by increased inflammation and oxidative stress. We also showed that the potent antioxidant and small-molecule GPx1 mimetic, ebselen, reduced plaque formation under diabetic conditions in both *ApoE^−/−^* and *ApoE/GPx1* dKO mice, via a mechanism that included modulation of the inflammatory MAPK, JNK and p38 pathways [276].

Furthermore, compounds like N-acetylcysteine (NAC), which serve as substrates for antioxidant enzymes, have been investigated for their potential to treat heart failure (NCT00532688). In patients with cardiorenal syndrome, NAC treatment was associated with improved forearm blood flow and significant improvements in endothelial function [277]. Similarly, natural antioxidants such as berberine and urolithin A decrease oxidative stress and improve endothelial function, thereby preventing the development of atherosclerosis and other heart-related diseases such as AMI [17,278,279].

The transcription factor Nrf2 acts as a master regulator of antioxidant signaling and maintains redox balance in cells. Multiple potential Nrf2 activators, including dimethyl fumarate (DMF), bardoxolone methyl, resveratrol, quercitol and curcumin, have been shown to modulate the pathophysiology of various CVDs [94,280]. DMF treatment reduced the levels of serum and aortic ROS, as well as the expression of the oxidation-related protein gp91^phox^, also known as Nox2. It upregulated the expression of HO-1 and Nrf2, thereby reducing aortic atherosclerosis in diabetic *ApoE*^−/−^ mice by activating the Nrf2/ARE signaling pathway [281]. Similarly, another potent Nrf2 activator, bardoxolone methyl, promoted Nrf2 binding to the transcriptional co-activator CREB-binding protein (CBP) and increased Nrf2 downstream targets, including NQO-1, HO-1, catalase and the glutamate-cysteine ligase catalytic (GCLC) subunit, consequently attenuating myocardial inflammation and improving cardiac function in rats with chronic heart failure [282].

These newer preclinical antioxidant strategies offer alternate approaches to the vitamin strategies previously used in clinical trials. These newer approaches are more likely to be efficacious as they overcome some of the limitations of vitamin therapy such as their lack of target specificity [283,284,285]. Data from the UK Biobank and FinnGen databases further highlight the inherent risks of vitamin therapy, where a recent study found that elevated levels of specific circulatory antioxidants, particularly α-tocopherol, α-carotene and retinol, were linked to increased risk of certain cardiovascular diseases [286]. Together, these insights underscore the urgent need for targeted, mechanism-based antioxidant therapies that not only avoid the pitfalls on non-specific vitamin supplementation but also hold genuine promise for reducing cardiovascular risk.

### 11.3. Therapies Targeting Mitochondrial Dysfunction and Autophagy in Cardiovascular Disease

Several therapeutic agents, including the AMPK activator metformin, CoQ10, MitoQ, the potent mitochondrial-targeted peptide SS-31 (also known as elamipretide) and the mitophagy inducer urolithinA, modulate multiple components of the redox–mitochondria–autophagy axis simultaneously. As discussed below, these agents are proving to be important modulators of CVD outcomes. For ease of reference, pharmacological inhibitors targeting redox signaling, mitochondrial dysfunction and autophagy in various cardiovascular conditions are listed in Table 1.

Importantly, therapeutic enhancements of pathways that control mitochondrial quality have shown beneficial effects in preclinical models of cardiovascular disease. Mitochondrial quality control involves multiple tightly coordinated processes, including mitochondrial biogenesis, fusion/fission dynamics, proteostasis and mitophagy. These processes collectively preserve mitochondrial function, control redox imbalance by regulating ROS generation and regulate cellular homeostasis.

#### 11.3.1. CoQ10

Coenzyme Q10 (CoQ10) or ubiquinone is an endogenously synthesized coenzyme and a key component of the ETC [287]. In particular, it is involved in the CoQ10–AMPK–OPA1 pathway, where it activates AMPK, which in turn upregulates OPA1, thereby enhancing mitochondrial function by promoting ATP production [27]. It reduces oxidative stress by decreasing levels of lactate dehydrogenase (LDH) and MDA, while increasing antioxidants such as SOD and GSH. It is therefore implicated as a therapeutic agent in the treatment of CVDs [27,287]. In preclinical studies, CoQ10 administered to a rat model of I/R injury ameliorated acute myocardial injury, reduced myocardial apoptosis and improved cardiac function by enhancing autophagy and reducing oxidative stress [288], suggesting that CoQ10 regulates redox signaling and improves mitochondrial function and autophagy to protect against CVD. A clinical study demonstrated that CoQ10 supplementation has potential prophylactic efficacy in reducing the incidence of fatal and non-fatal MI [289]. A systemic review and meta-analysis demonstrated that CoQ10 supplementation improved mitochondrial function, namely, ATP generation and respiratory capacity, and importantly, improved cardiovascular function, suggesting that CoQ10 could be a beneficial adjunct therapy for CVD patients [290].

#### 11.3.2. MitoQ

Mitochondria-targeted MitoQ plays a key role in mitochondrial quality control by triggering mitophagy, restoring mitochondrial membrane potential and improving mitochondrial dynamics [291]. In addition, MitoQ has shown promising antioxidant and anti-inflammatory effects in preclinical and clinical trials for various cardiovascular conditions by reducing mitochondrial ROS and improving mitochondrial function [268,292,293,294]. More specifically, MitoQ improved cardiac function by enhancing PINK1/Parkin-mediated mitophagy in Type 2 diabetic rats [29]. MitoQ also markedly reduced ROS levels and mitigated triptolide-induced cardiotoxicity by activating the autophagy p62-Nrf2 signaling pathway in H9c2 cardiomyocytes [293]. In a rat pressure overload-induced heart failure model, MitoQ significantly improved mitochondrial dysfunction by decreasing hydrogen peroxide, improving mPTP opening and enhancing mitochondrial respiration [291]. In addition, in a clinical trial of hypertensive patients, MitoQ supplementation administered together with moderate-intensity endurance training substantially reduced blood pressure and IL-6 levels and improved cardiac function in these patients [294]. One explanation involves the ability of MitoQ to downregulate MiR-21. Research has shown that MiR-21 is associated with ROS production, vascular remodeling, increases in the level of inflammatory C-reactive protein, and arterial stiffness [294,295]. In hypertensive patients, MitoQ treatment led to a significant reduction in circulating miR-21 levels, accompanied by improvements in LV mass and systolic function [294].

#### 11.3.3. Melatonin

Melatonin is a naturally occurring neurohormone primarily secreted by the pineal gland, and best known for its role in regulating the circadian rhythm and promoting sleep. Accumulating evidence now suggests that it exerts far-reaching protective effects beyond the brain, including the CV system. For example, in neonatal mouse ventricular cardiomyocytes subjected to hypoxia and reoxygenation injury, treatment with melatonin enhanced mitochondrial metabolism, inhibited mitochondrial oxidative stress, induced mitochondrial fusion and prevented mitochondria-driven apoptosis. Mechanistically, melatonin improved mitochondrial biogenesis by activating the AMPK/PGC1α pathway and attenuated I/R-induced myocardial damage [296]. Another study demonstrated that melatonin prevented the progression of atherosclerosis by inducing mitophagy and inhibiting activation of the NLRP3 inflammasome, which was mediated by the Sirt3/FOXO3a/Parkin signaling pathway [297]. Melatonin also suppressed galectin-3 (Gal-3), reduced the activity of the NF-κB signaling pathway and promoted the nuclear translocation of TFEB, thereby enhancing autophagy and suppressing inflammation in atherosclerosis. Furthermore, melatonin enhanced autophagy via inhibition of the Gal-3/CD98/PI3K pathway in THP-1 macrophages, and it alleviated inflammation, highlighting its potential as a therapeutic agent for the treatment of atherosclerosis [28]. Another study found that melatonin activated the autophagy process via the AMPK/mTOR/ULK1 signaling pathway and decreased vascular calcification of VSMCs isolated from the aortas of Sprague–Dawley rats [298]. Collectively, these data suggest a protective role for melatonin against cardiovascular injury and vascular calcification through modulation of autophagy.

#### 11.3.4. Urolithin A

Urolithin A is a regulator of mitophagy and exhibits cardioprotective effects [96,158]. Several studies have revealed that urolithin A upregulates the expression of mitophagy-related genes and activates PINK1-Parkin-mediated mitophagy, thereby improving mitochondrial quality control [96,158,299]. Impaired mitochondrial function is also a key feature of cardiac aging in humans. A recent study demonstrated that urolithin A improved cardiac function and mitochondrial health in aging mouse and rat models of heart failure with HFrEF. Urolithin A restored heart muscle ultrastructure and mitochondrial morphology, and it improved cardiac and skeletal muscle function in non-diseased old C57BL/6RJ mice. Moreover, urolithin A administered for 2 months improved systolic function, reduced the end-systolic volume and improved cardiac muscle contractility in rats with heart failure [30]. Mechanistically, in the hearts of AMI animals, urolithin A increased the levels of mitochondrial oxidative phosphorylation-associated genes, as well as the PINK1/parkin-mediated mitophagy marker, phospho-ubiquitin [30], suggesting that urolithin A exerts its cardioprotective effects by activating mitochondrial recycling and enhancing the mitochondrial quality control system [30]. In clinical trials, 4 months of urolithin A supplementation in healthy older adults significantly reduced plasma ceramide levels, which are associated with CVD risk in humans [30].

#### 11.3.5. Elamipretide

Mitochondria-targeted elamipretide (SS-31) has been shown to preserve mitochondrial dynamics and restore energy production in ischemic mitochondria by binding to and stabilizing cardiolipin [300]. Remarkably, elamipretide reversed age-associated post-translational modifications such as S-glutathionylation of cysteine residues and phosphorylation of heart proteins [301]. Moreover, SS-31 significantly suppressed mitochondrial ROS production, decreased protein oxidation and cellular senescence, improved cardiac function and mitigated myocardial hypertrophy in aged mice [302]. These results support the therapeutic potential of SS-31 in alleviating mitochondrial dysfunction and redox-driven pathologies in cardiovascular disorders, particularly in senescence.

#### 11.3.6. Metformin

Metformin, a widely used first-line therapy to improve glucose metabolism in type 2 diabetic patients, has emerged as a multifaceted agent that can simultaneously target redox signaling, mitochondrial function and autophagic flux, thereby maintaining cellular homeostasis [65,303]. A growing body of research demonstrates that metformin induces autophagy through multiple signaling pathways, including AMPK-dependent pathways such as AMPK/mTOR, AMPK/CEBPD, AMPK/ULK1 and AMPK/miR-221, where metformin directly and indirectly activates multiple autophagy-related proteins via inhibition of mTORC1 [304,305]. Additional autophagic pathways induced by metformin include Redd1/mTOR, STAT and SIRT, TRIB3 as well as the PK2/PKR/AKT/GSK3β pathway and the Na^+^/H^+^ exchangers [303]. Metformin also facilitates heart regeneration by enhancing autophagy in zebrafish [305]. Metformin has also shown protective effects against ischemic myocardial injury by reducing macrophage-driven inflammation via modulation of the autophagy–ROS–NLRP3 axis [65]. In Wistar rats, metformin reduced infarct size, cardiac arrhythmias and LV dysfunction by attenuating mitochondrial dynamic imbalance and apoptosis in cardiac ischemia–reperfusion injury, most likely mediated, in part, by the activation of the AMPK/PGC1α pathway [98]. However, clinical research investigating the use of metformin in CVD prevention remains limited and awaits more comprehensive and targeted studies to validate its cardioprotective potential.

#### 11.3.7. Berberine

Berberine, an isoquinoline alkaloid found in plants, has shown promising cardioprotective properties against different CVDs [306,307]. In mice with HFpEF, berberine upregulated p-AMPK and PGC-1α, reduced mtROS, improved mitochondrial function and alleviated mitochondrial biogenesis disorders, thereby improving cardiac function [308]. Oxygen–glucose deprivation/re-oxygen (OGD/R) of human cardiomyocytes inhibited the production of GSH, GPx and SOD and increased the production of MDA, IL-1β, TNF-α and IL-6, whereas treatment with berberine markedly reduced indicators of inflammation and oxidative stress. In both human cardiomyocytes and a myocardial I/R rat model, berberine ameliorated inflammation, oxidative stress and ischemia–reperfusion injury by inducing miR-26b-5p and suppressing the PTGS2/MAPK signaling pathway [279]. Furthermore, in an animal model of carotid atherosclerosis, berberine reduced the atherosclerotic plaque area, lipid accumulation, neointimal formation and cell apoptosis in carotid arteries by regulating the PI3K/AKT/mTOR signaling pathway, thereby improving carotid atherosclerosis [309]. A recent study further substantiated these findings in HFD-fed *ApoE*^−/−^ mice, where administration of berberine mitigated atherosclerosis by promoting autophagy, suppressing inflammatory responses and maintaining vascular endothelial cell integrity, via modulations of the RAGE-NF-κB pathway [278]. With many of these multifaceted effects mediated via AMPK activation, inhibition of pro-inflammatory signaling (e.g., NF-κB) and stimulation of Nrf2, berberine may offer an alternate approach to lessen CVDs, particularly as an adjunctive therapy for individuals with cardiometabolic disorders. However, numerous challenges remain regarding the formulation and clinical standardization [310,311].

**Table 1:** Pharmacological inhibitors targeting redox signaling, mitochondrial dysfunction and autophagy in CVDs.

Therapeutic Agent	Signaling Pathways and Related Mechanisms	Treatment Outcome	Experimental Models	Disease Context	Ref.
CoQ10	Inhibits oxidative stress. Improves mitochondrial function. Activates the AMPK-YAP-OPA1 pathway.	Increases SOD and GSH in serum in diseased mice. Suppresses the expression of IL-6, TNF-α, ICAM-1, VCAM-1 and NLRP3. Ameliorates atherosclerosis.	High-fat diet (HFD)-fed *ApoE*^−/−^ mice	Atherosclerosis	[27]
	Reduces oxidative stress. Enhances autophagy.	Increases GPx, GR, SOD and GSH. Decreases TBARS in myocardial tissue in rats with AMI. Increases autophagy proteins Beclin-1 and Atg5. Reduces infarct size. Improves cardiac function.	AMI/R Sprague–Dawley (SD) rat model	Acute myocardial ischemia–reperfusion injury (AMI)	[288]
MitoQ	Reduces oxidative stress. Activates p62-Nrf2 signaling pathway.	Decreases ROS accumulation. Improves cell viability. Reduces cardiotoxicity.	Triptolide-induced cardiotoxicity in rat cardiomyocyte H9c2 cells		[293]
	Decreases oxidative stress. Regulates mitochondrial function.	Restores mitochondrial membrane potential and respiration. Improves mitochondrial calcium retention capacity. Inhibits ROS production. Improves cardiac function.	Rat model of heart failure induced by pressure overload	Heart failure	[291]
	Enhances mitophagy via PINK1/Parkin pathway.	Reduces myocardial infarction, myocardial pathological damage and cardiomyocyte apoptosis. Improves cardiac function.	Myocardial ischemia–reperfusion injury in Type 2 diabetic rats	MIR injury in Type 2 diabetes (T2D)	[29]
Melatonin	Suppresses oxidative stress. Enhances mitochondrial biogenesis via the AMPK/PGC1α pathway.	Reduces mtROS production. Alters mitochondrial morphology of cardiomyocytes. Attenuates myocardial damage.	Hypoxia/reoxygenation injury in cardiomyocytes	Cardiac ischemia/reperfusion (I/R) injury	
	Reduces inflammation. Enhances autophagy. Promotes TFEB nuclear translocation. Inhibits NF-κB by inhibiting Gal-3.	Inhibits secretion of IL-6, IL-18, IL-1β and TNF-α in arteries. Inhibits atherosclerotic plaque progression.	HFD-fed *ApoE*^−/−^ mice	Atherosclerosis	[28]
Urolithin A	Restores mitochondrial dynamics proteins DRP1 and MFN1. Activates mitochondrial recycling and quality control (QC).	Improves heart mitochondrial ultrastructure, morphology and function. Enhances cardiac function and skeletal muscle force in aging.	Non-diseased old C57BL/6RJ mice	Aging	[30]
	Promotes mitochondrial QC pathways.	Improves systolic function. Improves cardiac function and mitochondrial health.	Rat model of chronic heart failure (HFrEF)	Heart failure	[30]
Elamipretide (SS-31)	Regulates age-associated post-translational modifications of heart proteins.	Affects mouse heart function.	Aged mouse hearts	Cardiac aging	[301]
	Suppresses mtROS production. Inhibits protein oxidation and cellular senescence.	Reduces cardiac hypertrophy. Improves cardiac function.	Aged mice	Myocardial hypertrophy	[302]
Metformin	Preserves mitochondrial function.	Alleviates mitochondrial dynamic imbalance and apoptosis. Reduces arrhythmia and infarct size. Improves cardiac function.	Cardiac I/R injury in Wistar rats	Cardiac ischemia/reperfusion (I/R) injury	[98]
	Induces autophagy.	Enhances epicardial, endocardial and vascular endothelial regeneration. Improves transient collagen deposition and resolution. Induces cardiomyocyte proliferation. Improves systolic function of the heart.	Adult zebrafish model of heart cryoinjury	Myocardial infarction	[305]
Berberine	Inhibits inflammatory responses and oxidative stress via miR-26b-5p-mediated PTGS2/MAPK.	Increases GSH, GSH-Px and SOD. Suppresses MDA, IL-1β, TNF-α and IL-6. Preserves myocardial structure. Improves cardiac function.	OGD/R-treated cardiomyocytes Rat model of myocardial ischemia-reperfusion (I/R) injury	Acute myocardial infarction model (AMI)	[279]
	Activates autophagy and reduces inflammation. Modulates RAGE-NF-κB.	Increases lipid accumulation and foam cell formation. Maintains vascular endothelial cell integrity. Reduces atherosclerotic inflammation.	High-fat diet *ApoE*^−/−^ mouse model	Atherosclerosis	[278]
	Regulates PI3K/AKT/mTOR.	Improves intimal hyperplasia. Reduces carotid lipid accumulation. Promotes cell proliferation.	High-fat diet *ApoE*^−/−^ mice	Carotid atherosclerosis	[309]
Mdivi-1	Suppresses mt-ROS/NLRP3 by inhibiting DRP1-dependent mitochondrial fission.	Decreases plaque area. Reduces foam cells. Inhibits M1 polarization. Inhibits activation of NLRP3.	High-fat diet *ApoE*^−/−^ mice	Atherosclerosis	[97]
DMF	Exerts antioxidant effects by activating the Nrf2/ARE signaling pathway.	Reduces the area of aortic atherosclerosis. Decreases serum and aortic ROS, HO-1, NF-κB, ICAM-1 and gp91phox. Increases serum and aortic Nrf2, eNOS and p-eNOS.	*ApoE*^−/−^ mice with streptozotocin-induced hyperglycemia	Atherosclerosis	[281]
Micheliolide (MCL)	Promotes KEAP1/Nrf2 dissociation. Activates Nrf2 pathway.	Decreases inflammatory responses. Reduces oxidative stress. Inhibits macrophage ferroptosis.	High-fat diet *ApoE*^−/−^ mice	Atherosclerosis	[105]
Bardoxolone- methyl	Increases Nrf2 binding to the CREB-binding protein. Increases Nrf2 downstream targets NQO1, HO-1 and CAT.	Reduces myocardial oxidative stress and lipid peroxidation. Attenuates myocardial inflammation.	Rat model of chronic heart failure	Chronic heart failure	[282]

## 12. Discussion

This review highlights the opportunity to target the interconnected redox–mitochondria–autophagy–inflammation axis for the prevention and treatment of CVDs. Significant progress has been made in the understanding of the role of redox processes in regulating mitochondrial function and autophagy, and its associated link with inflammation and immune responses.

However, one of the major challenges is maintaining context-dependent redox signaling, which if perturbed may contribute to the pathophysiology of CVDs. Depending on several factors, e.g., the cellular context or the stage of the disease, the impact on these pathways may not always be cardioprotective. Indeed, targeting specific redox signaling pathways may inadvertently modulate other signaling networks, triggering off-target and unwanted side effects. For example, multiple studies demonstrate that Mitochondrial Division Inhibitor 1 (Mdivi-1), which reversibly inhibits Complex I of the ETC to modify mtROS production [287,312,313], reduces atherosclerosis in *ApoE*^−/−^ mice [97]. However, mdivi-1 treatment in cardiomyocytes led to decreased OXPHOS complex protein expression, superoxide production and reduced mitochondrial respiration, resulting in functionally compromised mitochondria [314]. Another study suggested that apart from inhibiting Drp-1-mediated mitochondrial fission, mdivi-1 affected the ion channel function and altered the Rho kinase pathway, thereby affecting the regulation of vascular smooth muscle tone [315]. Thus, future studies should focus on therapies that target multiple nodes of the redox–mitochondria–autophagy–inflammation axis, with an emphasis on minimizing adverse effects.

Another major challenge for the field is the inability to accurately quantify the level of oxidative stress due to the high reactivity and relatively short half-life of reactive species. As a result, researchers must often rely on indirect measures or stable by-products of ROS to infer oxidative stress levels. Oxidative stress in humans can be measured during specific physiological and pathological states, such as metabolic stress conditions, acute ischemic events and post-reperfusion [316,317]. Human samples such as plasma, serum or urine can be used to assess the systemic redox status, whereas sampling specific cells or tissues, including leukocytes, endothelial cells or myocardial tissue samples, will allow the quantification of site-specific oxidative stress [316,317,318]. Several biomarkers of oxidative stress have been identified to evaluate oxidative damage and redox balance, including MDA and isoprostanes (IsoPs). Elevated levels of MDA and IsoP in biological fluids have significant clinical implications, as they represent a correlation with various cardiovascular risk factors and coronary artery disease [316]. 8-Hydroxy-2-deoxyguanosine (8-OHdG) has emerged as an important biomarker of oxidative damage of DNA, as guanosine is the most oxidized among the DNA nucleobases. A meta-analysis has demonstrated that elevated levels of 8-OHdG are observed in patients with CVD compared to healthy controls, suggesting that 8-OHdG may serve as a potential biomarker of oxidative DNA damage and CVDs [319]. Oxidative stress can also be evaluated by quantifying the total antioxidant capacity of human fluids using various assays such as the trolox equivalent antioxidant capacity assay (TEAC), the oxygen radical absorbance capacity assay (ORAC) and the ferric ion-reducing antioxidant power assay (FRAP) [316]. However, there are no generally accepted techniques to measure oxidative stress in clinical settings. Due to the dynamic nature of oxidative stress and the complexity of biological samples, a novel multifaceted approach may be required to accurately quantify oxidative stress in clinical practice. Advanced techniques, including metabolomics and proteomic approaches, can be used to profile oxidized metabolites and thiol modifications, which could offer insights into disease-specific redox regulation.

As discussed in this review, the integrity of the mitochondrial structure and function is key to maintaining redox homeostasis and mitigating the development of CVD. When considering the factors that contribute to CVD, the role that cellular senescence plays needs careful consideration. Age-related decline in mitochondrial mass contributes to excessive mtROS, with an associated depletion of ATP production. This drives endothelial and smooth muscle cell dysfunction, consequently contributing to CVDs such as atherosclerosis [200,238,320]. Removal of dysfunctional mitochondria should be a goal of CVD therapy. Indeed, the removal of dysfunctional mitochondria by enhancing mitophagy improves cardiac contractile function and retards cardiomyocyte senescence and remodeling of heart tissue [96,321,322]. In particular, treatment with tetrahydroberberrubine, a derivative of berberine, is showing promise in preclinical studies, where it promotes mitophagy in the aging heart, improves diastolic dysfunction, inhibits cardiac remodeling and suppresses cardiac senescence in aging mice [321]. Therefore, neutralizing excessive mtROS by targeted the delivery of ROS scavengers or improving mitochondrial function by enhancing mitochondrial biogenesis may offer alternative therapies for age-related diseases where mitochondrial dysfunction is causal [96]. Furthermore, targeting mitochondrial DNA, mitochondrial microRNAs and associated proteins offers compelling future directions for therapies aimed at restoring mitochondrial function [95].

Newer technologies may assist in delivering a more targeted approach to therapy. Emerging evidence demonstrates that targeted nanotherapeutics can improve therapeutic efficacy and reduce systemic adverse events and off-target effects in CVDs [323]. Nano-drug delivery systems can be engineered to specifically target the cells and/or cellular compartments within the heart or the vasculature, thereby allowing for precise therapeutic interventions. In particular, targeting impaired mitochondria within pathological tissue has emerged as a promising strategy [287,323]. Nanoparticle-based drug delivery systems can also be effective tools for targeting atherosclerotic plaque. For example, lipoic acid nanoparticles passively target atherosclerotic plaque and exhibit better therapeutic efficacy than free lipoic acid. Specifically, lipoic acid nanoparticles reduced oxidative stress and inflammation and inhibited lipid infiltration into plaques of HFD-*ApoE*^−/−^ mice [324], suggesting that nanoparticle-based drug delivery systems offer a promising therapeutic strategy for CVDs.

In addition, to identify patient subgroups most likely to benefit from specific interventions, integrative omics and bioinformatics approaches are likely to bolster therapeutic strategies. System biology approaches and multi-omics profiling can be used to identify dysregulated pathways linking redox imbalance, mitochondrial dysfunction, dysregulated autophagy and inflammation.

## 13. Conclusions

In summary, redox signaling, mitochondrial function, autophagy and inflammation form a complex interconnected network required for maintaining cellular homeostasis. Depending on the cellular and physiological context, these processes influence each other such that dysregulated redox balance impairs mitochondrial function, while dysfunctional mitochondria exacerbate maladaptive autophagy that further enhances inflammatory responses. Unless therapeutically targeted, chronic inflammation perpetuates mitochondrial dysfunction and impaired autophagy, inducing oxidative stress that, in combination, drives the progression of CVD. Clearly, perturbations such as hyperglycemia, dyslipidemia and hypertension dysregulate this axis, which contributes to the onset and progression of various cardiovascular conditions. In highlighting the interconnectivity of this axis, the present review has spotlighted a powerful avenue for therapeutic intervention in cardiovascular disease prevention and/or progression. Future research should focus on the identification of pharmacological interventions capable of modulating this axis. This would represent a promising and innovative approach to lessen the burden of CVDs.

### Novelty

This review unravels the interplay of redox signaling, mitochondrial dysfunction and the autophagy pathway in regulating cardiovascular inflammation and tissue damage in CVDs. This review contributes to a deeper understanding of the redox–mitochondria–autophagy–inflammation axis underlying CVDs. It summarizes recent findings and highlights the importance of focusing on redox pathways and associated signaling nodes to address the unmet clinical needs in CVD prevention.

## Data Availability

No new data were created or analyzed in this study. Data sharing is not applicable to this article.

## Abbreviations

The following abbreviations are used in this manuscript:

ACE	Angiotensin II-converting enzyme
AGEs	Advanced glycation end-products
AMI	Acute myocardial infraction
AMPK	AMP-activated protein kinase
ANT1	Adenine nucleotide translocase 1
ApoE^−/−^	Apolipoprotein E-deficient
ARBs	Angiotensin receptor blockers
AREs	Antioxidant response elements
ASC	Apoptosis-associated speck-like protein containing a CARD
ATM	Ataxia-telangiectasia mutated
BNIP3	Bcl-2/adenovirus E1B 19-kDa-interacting protein 3
CAD	Coronary artery disease
Caspase-1	Cysteinyl aspartate specific proteinase-1
CAT	Catalase
CBP	Transcriptional coactivator CREB-binding protein
circRNAs	Circular RNAs
CoQ10	Coenzyme Q10
CVD	Cardiovascular disease
CypD	Cyclophilin D
cytoROS	Cytosolic ROS
DCM	Diabetic cardiomyopathy
DMF	Dimethyl fumarate
DMPs	Damage-associated molecular patterns
DRP1	Dynamin-related protein 1
ER	Endoplasmic reticulum
Gal-3	Galectin-3
GCLC	Glutamate cysteine ligase catalytic
GeX1	Gerontoxanthone I
GPx	Glutathione peroxidase
GR	Glutathione reductase
GSH	Glutathione
GSNOR	S-nitrosoglutathione reductase
GSSG	Glutathione disulfide
H_2_O_2_	Hydrogen peroxide
H_2_S	Hydrogen sulfide
HAECs	Human aortic endothelial cells
HFD	High-fat diet
HFpEF	Heart failure with preserved ejection fraction
HIF-1α	Hypoxia-inducible factor-1α
HO-1	Heme oxygenase-1
Hsp70	Heat shock protein 70
I/R	Ischemia–reperfusion
IKKβ	Inhibitor kappa-B kinaseβ
IL-1β	Interleukin-1β
IL-6	Interleukin-16
LC3	Microtubule-associated protein 1A/1B-light chain 3
LDH	Lactate dehydrogenase
LDL	Low-density lipoprotein
LKB1	Liver kinase B1
LOX-1	Lectin-like oxidized LDL receptor
LTF	Lactoferrin
LV	Left ventricular
MAO-A	Monoamine oxidase A
MAPK	Mitogen-activated protein kinase
MCL	Micheliolide
MCP-1	Monocyte chemotactic protein-1
MCU	Mitochondrial calcium uniporter
McX	Macluraxanthone
MD1	Myeloid differentiation protein 1
MDA	Malondialdehyde
Mdivi-1	Mitochondrial division inhibitor 1
MFN1	Mitofusins 1
MFN2	Mitofusins 2
MI	Myocardial infarction
Mito-Esc	Mitochondria-targeted esculetin
MMPs	Matrix metalloproteinases
mPTP	Mitochondrial permeability transition pore
MsrA	Methionine sulfoxide reductase A
mtDNA	Mitochondrial DNA
mtKATP	Mitochondrial adenosine triphosphate (ATP)-sensitive potassium K channel
mTOR	Mechanistic target of rapamycin
mtROS	Mitochondrial ROS
NAC	N-acetylcysteine
NEK7	NIMA-associated kinase 7
NF-κB	Nuclear factor kappa B
NLRP3	Nucleotide-binding domain, leucine-rich-containing family, pyrin domain-containing-3
NO	Nitric oxide
NO_2_	Nitrogen dioxide
NOS	Nitric oxide synthases
NOX	NADPH oxidase
Nrf2	Nuclear factor erythroid 2–related factor 2
Nt-R	N-terminal arginine
^1^O_2_	Singlet oxygen
O_2_^•−^	Superoxide
O^8^G	8-oxoguanosine
^•^OH	Hydroxyl radicals
OGD/R	Oxygen–glucose deprivation/re-oxygen
ONOO^−^	Peroxynitrite
OPA1	Optic atrophy 1
ox-LDL	Oxidized low-density lipoprotein
OXPHOS	Oxidative phosphorylation
PAI-1	Plasminogen activator inhibitor-1
PDE5	Phosphodiesterase 5
Prx	Peroxiredoxin
RNS	Reactive nitrogen species
ROMO1	Reactive oxygen species modulator 1
ROS	Reactive oxygen species
RSS	Reactive sulfur species
RV	Right ventricular
Sirt1	Sirtuin 1
SLC26A4	Solute carrier family 26 member 4
SO_2_	Sulfur dioxide
SOD	Superoxide dismutase
SR-1	Scavenger receptor-1
STZ	Streptozotocin
T2D	Type 2 diabetes
TAC	Transverse aortic constriction
TFEB	Transcription factor EB
TIMPs	Tissue inhibitors of metalloproteinases
TLR	Toll-like receptor
TPP^+^	Triphenylphosphonium cation
Trx	Thioredoxin
TRXox	Oxidized thioredoxin
TRXred	Reduced thioredoxin
ULK1	Autophagy-activating kinase 1
Vcp	Valosin-containing protein
VDAC1	Voltage-dependent anion channel 1
VSMCs	Vascular smooth muscle cells
XO	Xanthine oxidase
XOR	Xanthine oxidoreductase
ZDHHC13	Zinc finger DHHC-type palmitoyltransferase 13
ΔΨm	Mitochondrial membrane potential
